# Evaluating the fishery and ecological consequences of the proposed North Sea multi-annual plan

**DOI:** 10.1371/journal.pone.0190015

**Published:** 2018-01-02

**Authors:** Steven Mackinson, Mark Platts, Clement Garcia, Christopher Lynam

**Affiliations:** 1 Centre for Environment, Fisheries and Aquaculture Science, Lowestoft, Suffolk, United Kingdom; 2 Ardent Data Analytics, Sheffield, South Yorkshire, United Kingdom; Sveriges lantbruksuniversitet, SWEDEN

## Abstract

The possible impacts of the European Commission’s proposed North Sea Multi-Annual Plan are evaluated in terms of its likely outcomes to achieve management objectives for fishing pressure, species’ biomass, fishery yield, the landed value of key species and ecosystem objectives. The method applies management strategy evaluation procedures that employ an ecosystem model of the North Sea and its fisheries as the operating model. Taking five key dimensions of the proposed plan, it identifies those areas that are key to its successful performance. Overwhelmingly, choices in the options for the implementation of regulatory measures on discarding practices outweigh the effects of options related to fishing within ranges associated with ‘pretty good yield’, the way that biomass conservation safeguard mechanisms are applied and the timeframe for achieving fishing mortality targets. The impact of safeguard options and ranges in fishing mortality become important only when stock biomass is close to its reference points. The fifth dimension–taking into account wider conservation and ecosystem objectives—reveals that discard policy has a big impact on conservation species, but also that the type of harvest control rule can play an important role in limiting risks to stocks by ‘applying the brakes’ early. The consequences to fisheries however is heightened risk to their viability, thus exposing the sustainability trade-offs faced with balancing societal pressures for blue growth and enhanced conservation. It also reveals the wider ecosystem impacts that emphasise the connectivity between the demersal and pelagic realms, and thus, the importance of not treating the demersal NSMAP in isolation from other management plans. When stocks are below their biomass reference points, low F strategies lead to better long term economic performance, but for stocks consistently above biomass reference points, high F strategies lead to higher long term value. *Nephrops* and whiting often show contradictory responses to the strategies because changes in their predators abundance affects their abundance and success of their fisheries.

## Introduction

Without additional legislation, the Basic Regulation of Europe’s Common Fisheries Policy (CFP) [1] could lead to under-utilisation of quota in the North Sea mixed fisheries if the implementation of the landing obligation lead to choke species (i.e. species whose catches quickly exceed available quota), halting fishing activity as a result. Faced with the risk of dis-agreeable and unworkable management that this implies, the European Commission (EC) has taken the initiative to propose a mixed fisheries multi-annual managemement plan for demersal species in the North Sea [2]; the objective being to acheive sustainable exploitation of principal demersal stocks by exploiting them according to the principles of maximum sustainable yield (CFP Article 2.2) and the ecosystem approach to fisheries management (CFP Article 2.3). The plan is also intended to facilitate the introduction of the landing obligation (CFP Article 15) through measures to eliminate unwanted discarding of commercial stocks. Solving the choke species problem is implicit in this objective.

We evaluated the performance of alternative strategies for implementation of the proposed North Sea multi-annual plan (NSMAP) using a multi-species, multi-fleet ecosystem model. Following the definitions in the proposed NSMAP, the alternative strategies were specified according to the ranges of F_msy_ (i.e. fishing rates associated with ‘pretty good yields’; [3,4,5,6], safeguard mechanisms, discard policy and application of F_msy_ for other assessed species in the North Sea—in accordance with MSY policy, Article 2.2 of the CFP.

The performance of the strategies was measured by their ability to meet regulatory targets for sustainable exploitation (F_msy_, total allowable catches, eliminating discards), their effect on resource conservation targets (stock biomass relative to biological reference points), their economic performance (fisheries value) and their risk to conservation species and ecosystem effects (Good Environmental Status (GES) indicators of food-webs). Consideration of these multiple objectives classifies the evaluation as taking a postmodern view of sustainability [7], aiming to contribute to the progress in developing more comprehensive evaluations of management plans (*Sensu* Trenkel et al. [8]).

We used a bespoke routine developed inside the ecosystem modelling framework Ecopath with Ecosim (EwE) [9,10,11], specifically designed to assess the robustness of management strategies to the uncertainties in model predictions arising from model error, observation error, and implementation error [12]. Core to the routine is the Management Strategy Evaluation (MSE) methodology [13,14,15], which addresses the need to provide decision makers with the risks and uncertainties associated with alternative strategies, and thus identify where trade-offs may lie. Within MSE, an operating model which represents a realisation of a biological model for the ‘real world’ is used as the platform for testing the performance of alternative management strategies.

The purpose of using a multi-species, multi-fleet model as the operating model in this impact assessment exercise is to be able to simultaneously take in to account the interactions among species and among fleets, thus making it possible to diagnose possible ecological and fishery trade-offs resulting from both direct and indirect fishing and food-web effects. This is particularly pertinent in light of the commitment of the CFP to implement regional sea-basin management (Article 18) based on an ecosystem approach. Articles 9 and 10 provide the basis for establishment of multi-annual plans; stating that they shall cover ‘…*in the case of mixed fisheries or where the dynamics of stocks relate to one another*, *fisheries exploiting several stocks in a relevant geographical area*, *taking into account knowledge about the interactions between fish stocks*, *fisheries and marine ecosystems*’. This commitment underpins the salience of this evaluation of the proposed multi-annual plan for mixed demersal fisheries in the North Sea.

Because ecology dictates that the collateral impacts of the proposed NSMAP will extend beyond the demersal species that it is focussed upon, the wider ecosystem and fishery consequences also need to be brought in to the light for decision making. Again, this is particularly pertinent now because integration of fisheries and environmental policy is front and centre in shaping research approaches (e.g. [16]) and delivery of integrated scientific advice in Europe [17, 18, 19] and beyond. In particular, we looked at the food-web indicators relevant to determining GES [20, 21] and impact on the pelagic species and fisheries as reflection of intimately linked dynamics between the demersal and pelagic realms of the North Sea ecosystem [22, 23].

## Materials and methods

### Process overview

**Fig 1:**
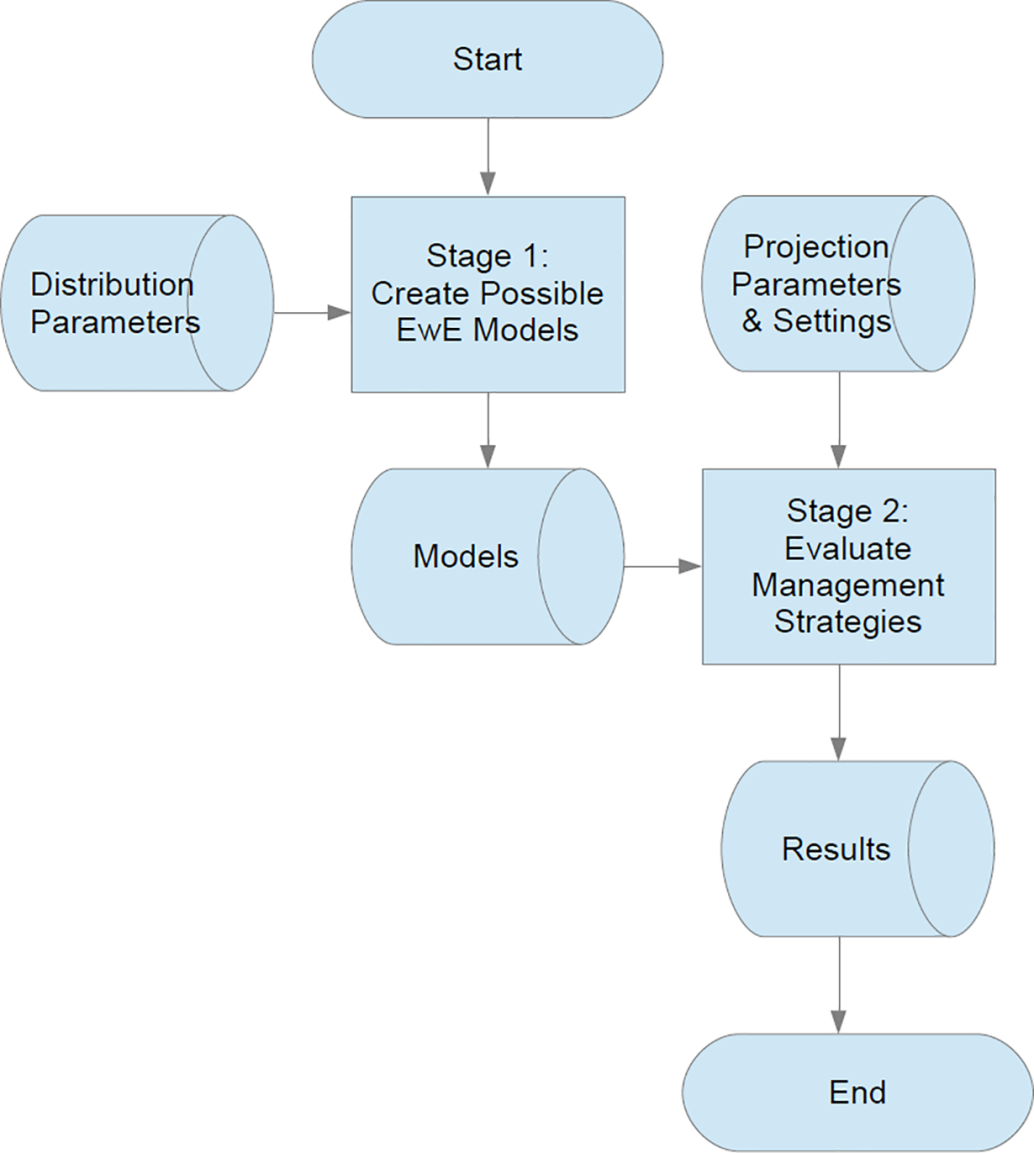
Flow chart showing an overview of the two main stages of the plugin.

**Fig 2:**
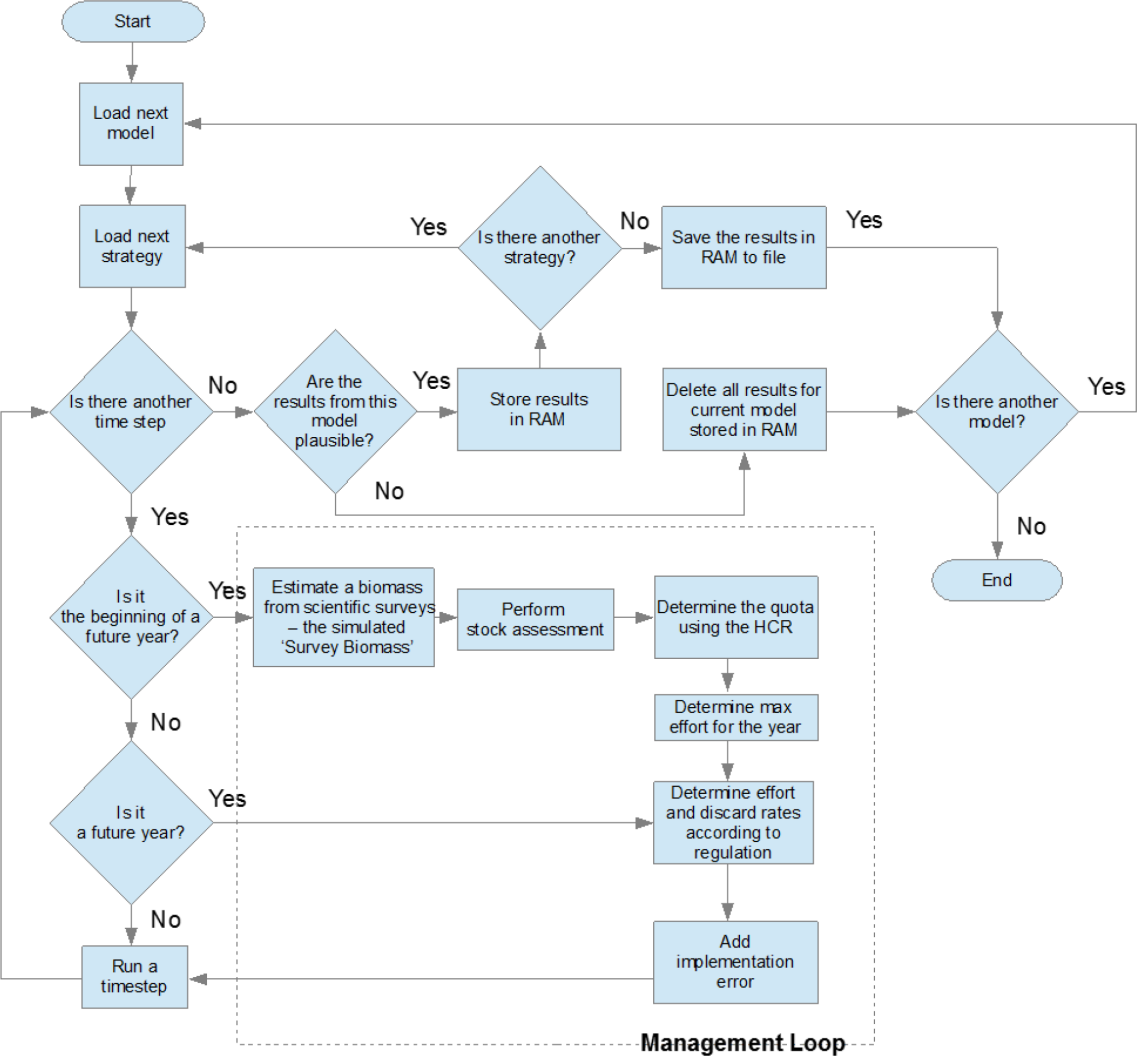
Flowchart for the evaluation of management strategies.

The procedure of the routines used to assess the performance of the management strategies and their robustness to uncertainties in the model predictions depicts a 2 stage process (Fig 1): stage 1 generates multiple plausible versions of the ecosystem model by sampling distributions of input parameters (biomass, feeding, production and consumption rates and predator-prey interaction rates), stage 2 simulates the application of alternative management strategies—defined by their harvest control rules and regulatory mechanisms—to each of the plausible models generated in stage 1 for the specified period (Fig 2). Upper and lower biomass bounds based on long term biomass trends are used to screen out the strategies whose trajectories are unrealistic. Full technical details of the routines are reported in Platts and Mackinson (2017, [12]), and the supporting information describes the key elements necessary to understand how the alternative strategies were implemented and behave in the model.

### Operating model(s)

For stage 1, the 2015 Key Run of the North Sea ecosystem model [24] was resampled to generate 1000 alternative plausible operating models for the management strategy evaluation. The Key Run is parameterised for the period 1991–2013 using stock assessment and survey data from the area defined in the NSMAP. It includes 69 functional groups and 10 fishing fleets. Fish groups subjected to stock assessment are specified at the species level, and international fleet segments are specified according to level 2 definitions of the EU Data Collection Framework. Economic data on fish prices and fleet costs from the 2015 EU Annual Economic report [25] were included in the model to support the evaluation of economic performance based on recent data.

While the historical simulation of the model is conditioned with the inclusion of environmental time series such as temperature, it is currently not possible to include these effects in forward simulations used in the evaluation of harvest strategies. Therefore, environmental conditions are assumed to remain constant from 2013 onward and their effects to apply equally to all the harvest strategies; this places the focus of the analysis on the contrast in performance among them.

### Species and their reference points for management and conservation (Table A in S1 Supporting information)

The analysis centres on the demersal species in the North Sea (cod, haddock, saithe, whiting, plaice, sole—defined as Group 1 in the NSMAP—and *Nephrops*—Group 2 -). We defined the lower, middle and upper ranges of fishing mortality, and the biomass target (B_msy_) and limit (B_lim_) values defined in Annex 1 & 2 of the proposed NSMAP as the management and conservation reference points. In the case of whiting, which has undefined reference points, we used F_lower_ = 0.14, F_target (msy)_ = 0.15 and F_upper_ = 0.33 based on ICES (2014), and B_msy_ = 250 kt, B_lim_ 184 kt based on Table 10.5.1 in ICES (2012, [26]). For *Nephrops*, we calculated an average of the functional units in the Annexes and converted the abundance to biomass based on a mean weight published in the International Council for Exploration of the Sea (ICES) *Nephrops* working group report [27].

To fully represent the CFP objectives on implementation of MSY policy and to take account of the fishery and ecosystem interactions that affect the proposed NSMAP, we included fishing and conservation reference points for other assessed demersal species subject to catch limits (Group 3, 7), as well as pelagic species (herring, mackerel, sprat, sandeels and horse-mackerel). Finally, we included conservation reference targets for prohibited elasmobranchs (Group 6) which were utilised in a particular set of strategies.

For species subject to catch limits but for which no reference points were available, we used the ecosystem model to estimate internally consistent reference points that could be used to calculate the Total Allowable Catch (TAC) and fleet quotas for each of the alternative management strategies. Using the model to calculate the equilibrium biomass and catch associated with fishing mortalities from zero to 5 times initial F (see [28]), we recorded F_msy_ and defined B_lim_ = 20% of the unfished biomass for each species. We set F_lower_ and F_upper_ equal to F_msy_, and set B_msy_ equal to the next highest thousand above B_lim_. We consider this approach to be acceptable given that the NSMAP states ‘*In the absence of scientific advice on fishing mortality rates consistent with maximum sustainable yield*, *fishing opportunities shall be consistent with scientific advice to ensure the sustainability of the stocks in line with the precautionary approach*.’ Note that a Precautionary Approach (PA) is described in the UN Fish Stocks Agreement (UN, 1995) as follows: “*States shall be more cautious when information is uncertain*, *unreliable or inadequate*. *The absence of adequate scientific information shall not be used as a reason for postponing or failing to take conservation and management measures*.” This implies that as information becomes increasingly limited and/or less certain, ICES advice on management will be more conservative with respect to possible impact on the marine ecosystem [29].

### Management strategies

**Fig 3:**
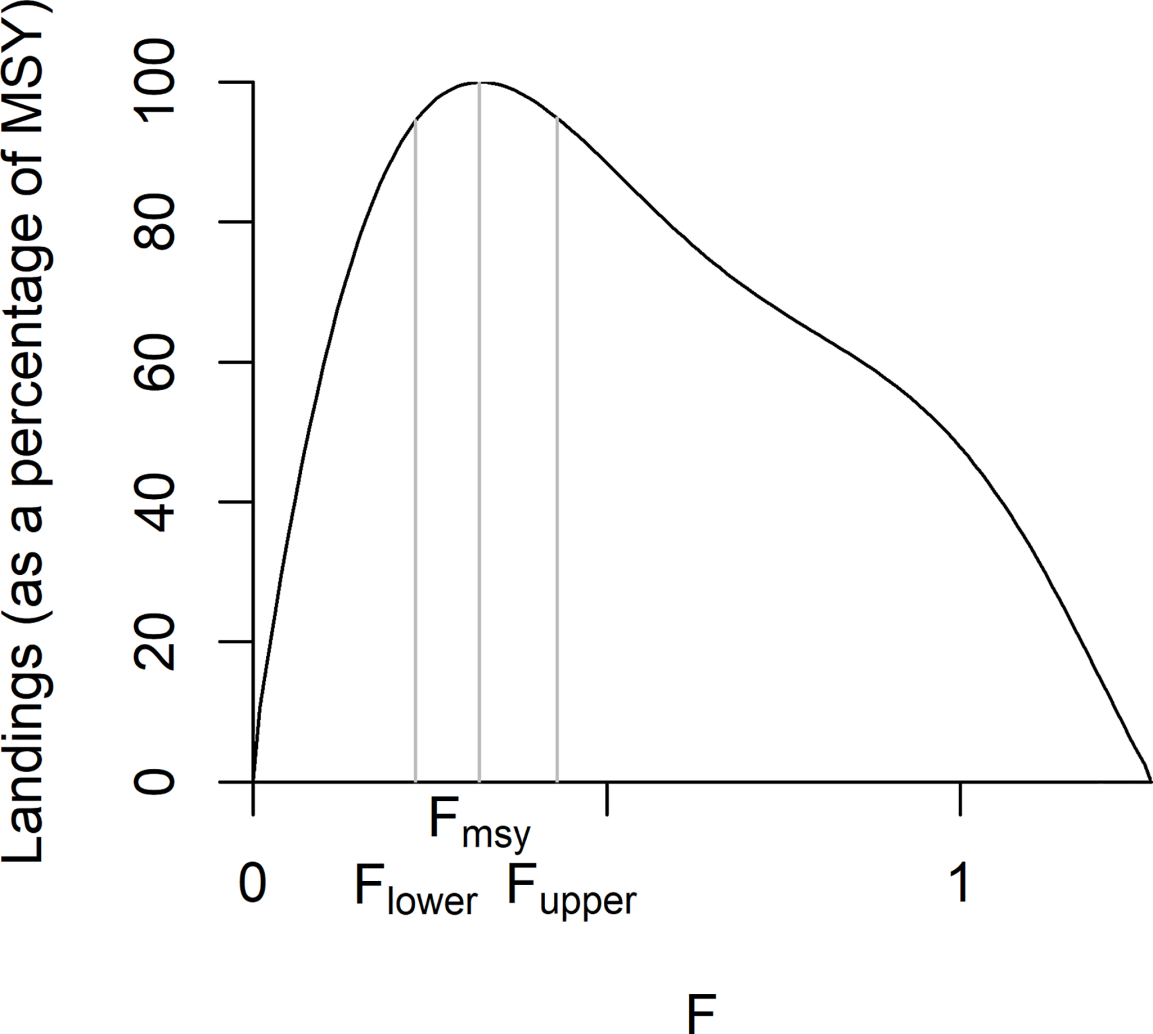
Example of a curve showing landings (as a percentage of MSY) as a function of fishing mortality (F). The location of F_msy_, F_lower_ and F_upper_ are indicated, the latter two corresponding to 95% of the peak of the median landings curve. Units are standardised to a scale of 0–100.

Alternative management strategies (Table B in S1 Supporting Information) were configured based on different choices along the five key dimensions specified in the proposed NSMAP:

**1. Contributing to the elimination of discards for species subject to catch limits**
Three scenarios on discard regulation were considered. Both “strict no discarding” and “avoiding discarding” represent no discard policies, differing only in the way they represent two extremes among the range of possibilities of fleet responses. Continued discarding represents a scenario of discarding akin to former years. In summary the three scenarios are:
  - **Strict no discarding**. Fishing stops when the quota of the weakest stock is exhausted. (*Note*: this is referred to as the minimum option under ICES mixed fisheries scenarios).
  - **Avoiding discarding**. Fisheries are able to take measures to fish more selectively, avoiding catching any fish whose quota is exhausted while continuing to fish for those that remain.
  - **Continued discarding**. Fishing continues until all the quotas are taken. Any fish caught over quota are discarded. (*Note*: this is referred to as the maximum option under ICES mixed fisheries scenarios).
  - Technical details of each of these scenarios application in the evaluation routine are given in the supplementary material.
**2. Flexibility to set the F on each species within a range**
  (see above and Table A in S1 Supporting Information)
  For each species, ranges in the fishing mortality reference points were taken from values as defined in Annex 1 & 2 of the proposed NSMAP. Terminology used in the modelled strategies is such that F_lower_ = F_low_, F_middle_ = F_target_ = F_msy_, and F_upper_ = F_high_. (Fig 3). The strategies explore the contrast when the target F for all species are applied simultaneously at either their lower, middle or upper values for the duration of the simulation. This represents a test of the sensitivity to the effects of the minimum and maximum conditions, which is suitable for assessing the impacts of changes in target F relative to those of other dimensions.

**Fig 4:**
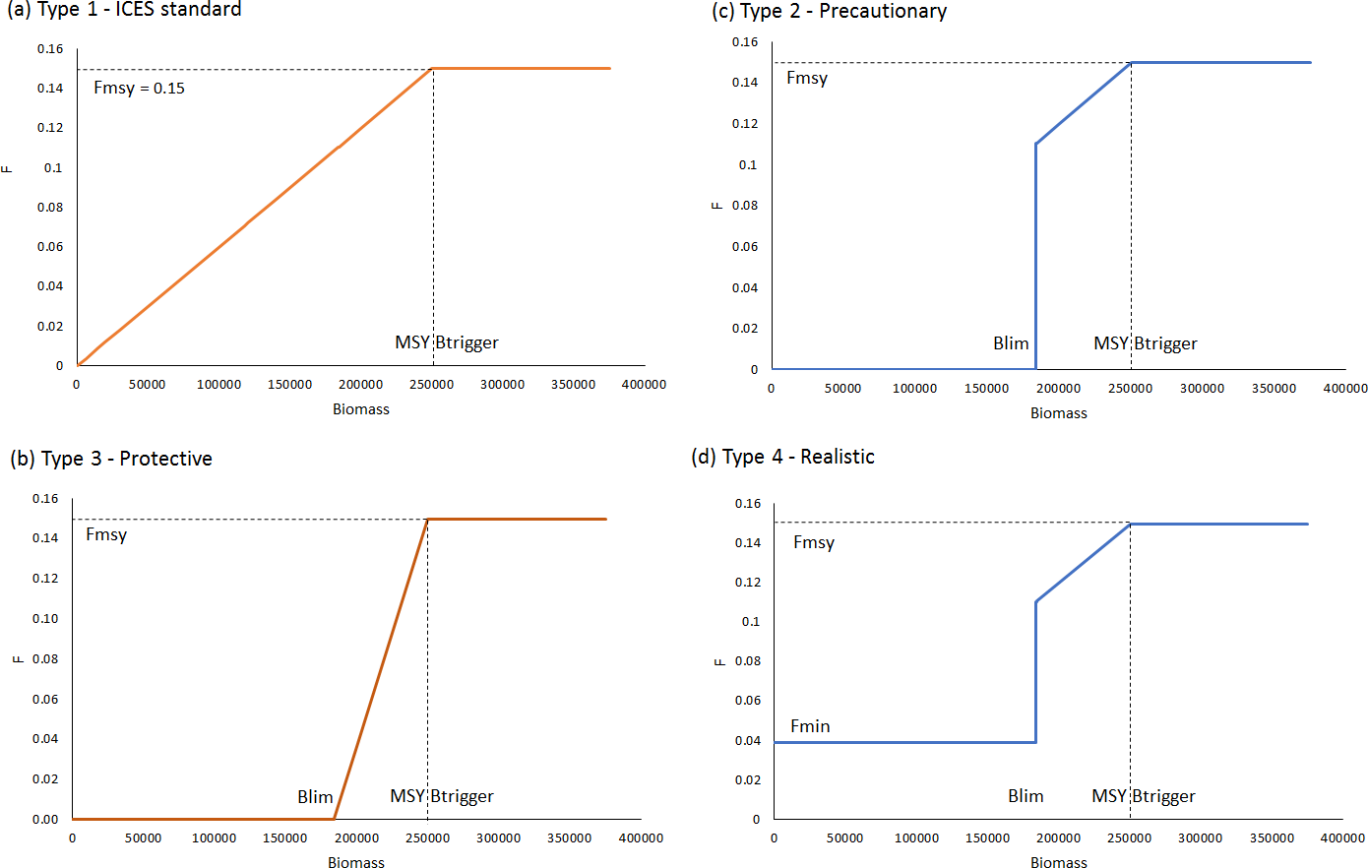
HCR types used in defining alternative strategies.

**3. Allowing for safeguards, whereby fishing pressure is reduced if stocks fall below safe biological limits**
  The way safeguards are applied to control the rate of fishing mortality at given stock biomass levels is called a Harvest Control Rule (HCR), or Advice Rule in ICES terminology. Four types of HCRs that are consistent interpretations of the definitions in the proposed NSMAP, were considered in the evaluation (Fig 4).
  **Type 1** is the **ICES standard** advice rule–where F declines linearly to zero when biomass is below MSY B_trigger_.
  **Type 2** is **Precautionary,** easing the rate of reduction in F between MSY B_trigger_ and B_lim._ The slope is initially equivalent to that in Type 1, however, at B_lim_ immediate action is taken to stop targeted fishing, with the result that F = 0 at and below B_lim_.
  **Type 3** is most **Protective**, applying the most severe reductions in F, which declines linearly from F_msy_ at MSY B_trigger_ to zero at B_lim_ (i.e. with a steeper slope than in Type 1 and 2).
  **Type 4** is considered the most **Realistic**, similar to Precautionary, but recognises that a small level of residual non-target by-catch mortality may remain on a stock at B ≤ B_lim_ even once those fleets targeting the stocks have been stopped from fishing.

**4. Time to achieve Fmsy**
The proposed NSMAP states ‘The target fishing mortality shall be achieved as soon as possible, and on a progressive, incremental basis by 2020 for the stocks of Groups 1 and 2.’ We compared two options for the timing of adjusting fishing mortality to achieve F_msy_ when the current F is higher than the target F. Option 1 adjusts F in equal yearly steps to achieve F_msy_ by 2015, Option 2 adjusts F in equal yearly steps so that F_msy_ is achieved by 2020.
**5. Contribute to implementation of the ecosystem-based approach to fisheries management**
  The proposed NSMAP has the specific objective to implement the ecosystem-based approach to fisheries management in order to ensure that negative impacts of fishing activities on the marine ecosystem are minimised. In particular, it stresses that this should be coherent with EU environmental legislation objectives of achieving GES by 2020 as set out in Article 1(1) of Directive 2008/56/EC.
  To consider the wider effect of the plan on biodiversity, we evaluated a strategy specifically designed to address objectives for the conservation of protected skates and ray and we investigated how choices in the other dimensions affected the outcomes of this strategy. In this ‘conservation’ strategy, conservation HCRs (see supplementary material) were used to override the setting of fishing rates on commercial stocks if the biomass of any conservation species falls below a lower threshold, which we set at 20% of the unfished model biomass determined from equilibrium simulations. The effect is that fishing rates on target species become contingent upon the biomass of the conservation species.
  A second conservation strategy, called ‘cod god’ uses the same principles as applied in the skates and rays conservation strategy but puts the management of cod above all other stocks with conservation measures on all fished species activated when cod biomass reaches its limit reference point, B_lim_. This mimics the former cod-recovery plan where increasingly restrictive measures were put in place as the cod stock declined. The purpose of this strategy was to examine the impact of cod management measures on the other stocks and fisheries, as well as to see the effect on cod recovery.
  We also investigated the broader ecosystem impacts of the strategy options on food webs by looking at their effects on GES indicators of change in the fish community.

Each strategy was simulated forward for 20 years in each of the 1000 possible operating models of the North Sea ecosystem, and their performance measured in relation to:

- The efficacy of management in terms of the contrast between the fishing mortality realized by the management strategy versus that set by the HCR. Note that fishing mortality associated with landings (landed F) was accounted for separately from that due to discarding (discard F). Choke species whose quota limited the effort of fleets under the strict no discard option were recorded.
- The biomass of the target stocks relative to their conservation reference points.
- The fishery performance, measured in terms of value to the demersal fleet (Demersal trawl and seine, beam trawlers, *Nephrops* trawlers), pelagic fleet and overall.
- The risk to conservation species, any species in groups 3 to 7 that have conservation reference limits breached and thus require remedial action (see Article 9, p19).
- The ecological impact on the marine ecosystem measured in relation to objectives for achieving GES. In particular, performance of indicators for Descriptors D1 biodiversity and D4 food webs were quantified.

Based on the combinations among the 5 dimensions, more than 60 different strategies were evaluated in total, each providing results for 69 functional groups and 11 fleets—amounting to 10s of gigabytes of output. To draw out the salient features from the contrast among the 5 dimensions and their relevance to the species and fleets in the proposed NSMAP, the analysis below distils the results from the 10 main strategies described in Table B in S1 Supporting information.

## Results

Comparison between the five key dimensions of the proposed NSMAP strategies show overwhelmingly that the choice of discard regulation (dimension 1) has the largest impact on the contrast in performance among the strategies. The weakest impact is related to the time to achieve F_msy_ (dimension 4). Allowing flexibility in the range of target fishing (dimension 2) and alternative configuration of safeguard options (dimension 3) have detectable but small effects, the impact of which becomes more apparent when stock biomass is close to its reference points. The fifth dimension reveals that discard policy has a substantial impact on conservation species and fishery sustainability. The type of harvest control rule implemented can play an important role in limiting risks to species by ‘applying the brakes early’ but such an approach may lead to greater risk to fishery sustainability. It also reveals the wider community impacts that emphasise the connectivity between the demersal and pelagic realms of the ecosystem.

**1. Contributing to the elimination of discards for species subject to catch limits**

**Fig 5:**
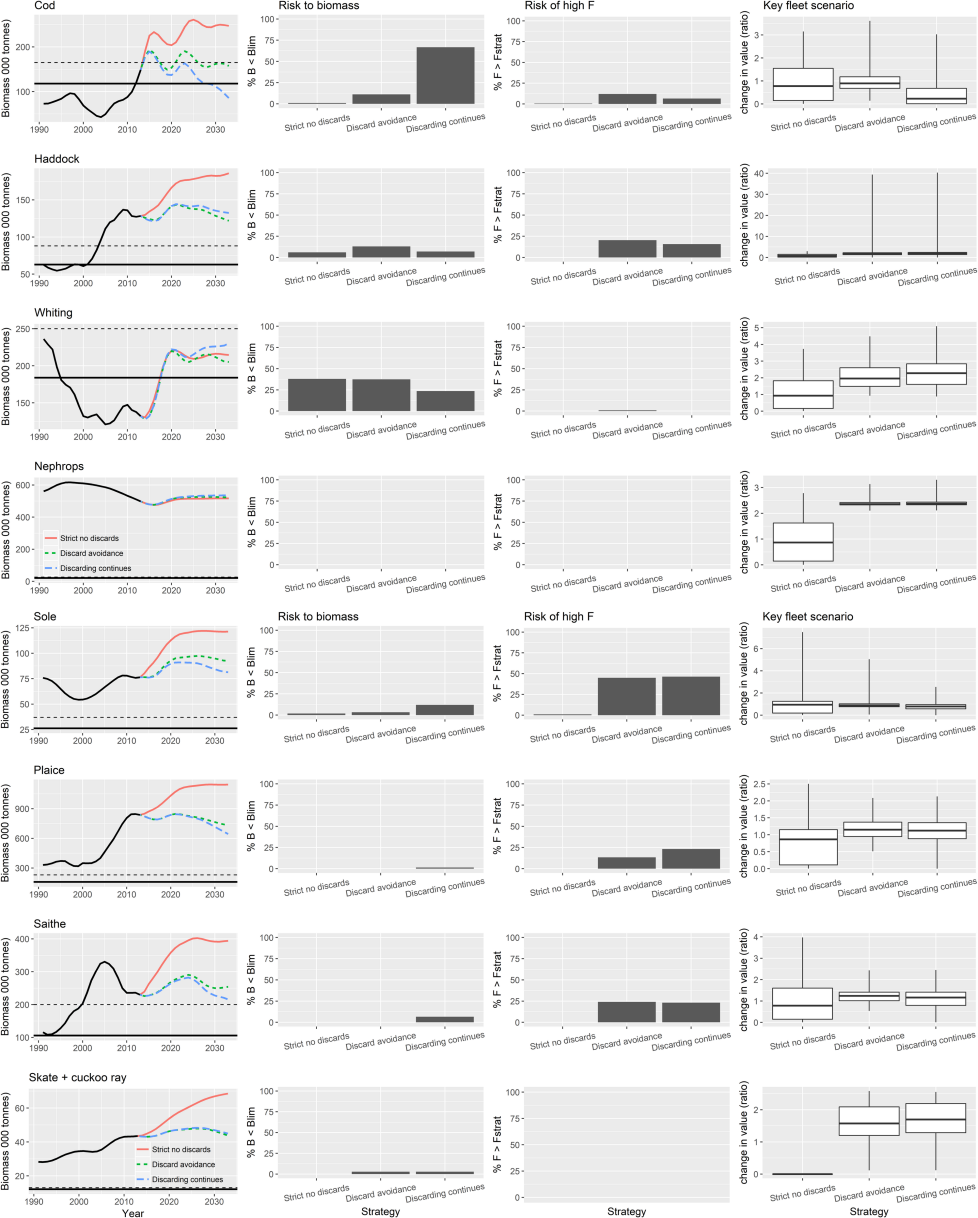
Contrast between 3 NSMAP strategies with safeguard using Realistic HCR type 4 (see Fig 4) and fishing at F_msy_ by 2020 in each. Strict no discards (red and left bar/box in each pane), discard avoidance (green and middle bar/box), discard continues (blue and right bar/box). The solid black line in the first column of plots shows B_lim_ and dotted line MSY B_Trigger_. The plots of risk of high F show the proportion of simulations (but do not reflect the magnitude) with landed F> the target F defined by the harvest control rule used in the strategy.

For cod, only the strict no discard policy leads to the projected stock recovery above MSY B_trigger_ (Fig 5). Discard avoidance strategies are projected to maintain biomass consistently above B_lim_ (~10% risk of B < B_lim_) and on average close to MSY B_trigger_, while continued discarding leads on average to a long-term decline in biomass with a high risk (>60% of B < B_lim_). Continued discarding means that management targets for F are entirely ineffective, because although the plots show that there is a limited risk of a landed F value being greater than the target F (<10%), and (somewhat confusingly) this is lower than the risk of landed F> target F under discard avoidance, when discard F is included, the total realized mortality is in fact above the target F in almost every simulation. Looking across the other species in Fig 5, there are other examples where the risk that landed F exceeding target F is similar under discard avoidance and continued discarding. However, this risk represents the frequency of exceptions rather than the magnitude which is always greater under continued discarding. The exceptions arise as a consequences of representation of the uncertainties in the model expressed as implementation errors.

Similar patterns are seen in the other species, with the exception of *Nephrops* and whiting that respond in the opposite way. Here the indirect effects that emerge from the predator-prey relationships in the ecosystem model are seen. They have an impact both on the stock status and performance of fisheries that target them.

**Fig 6:**
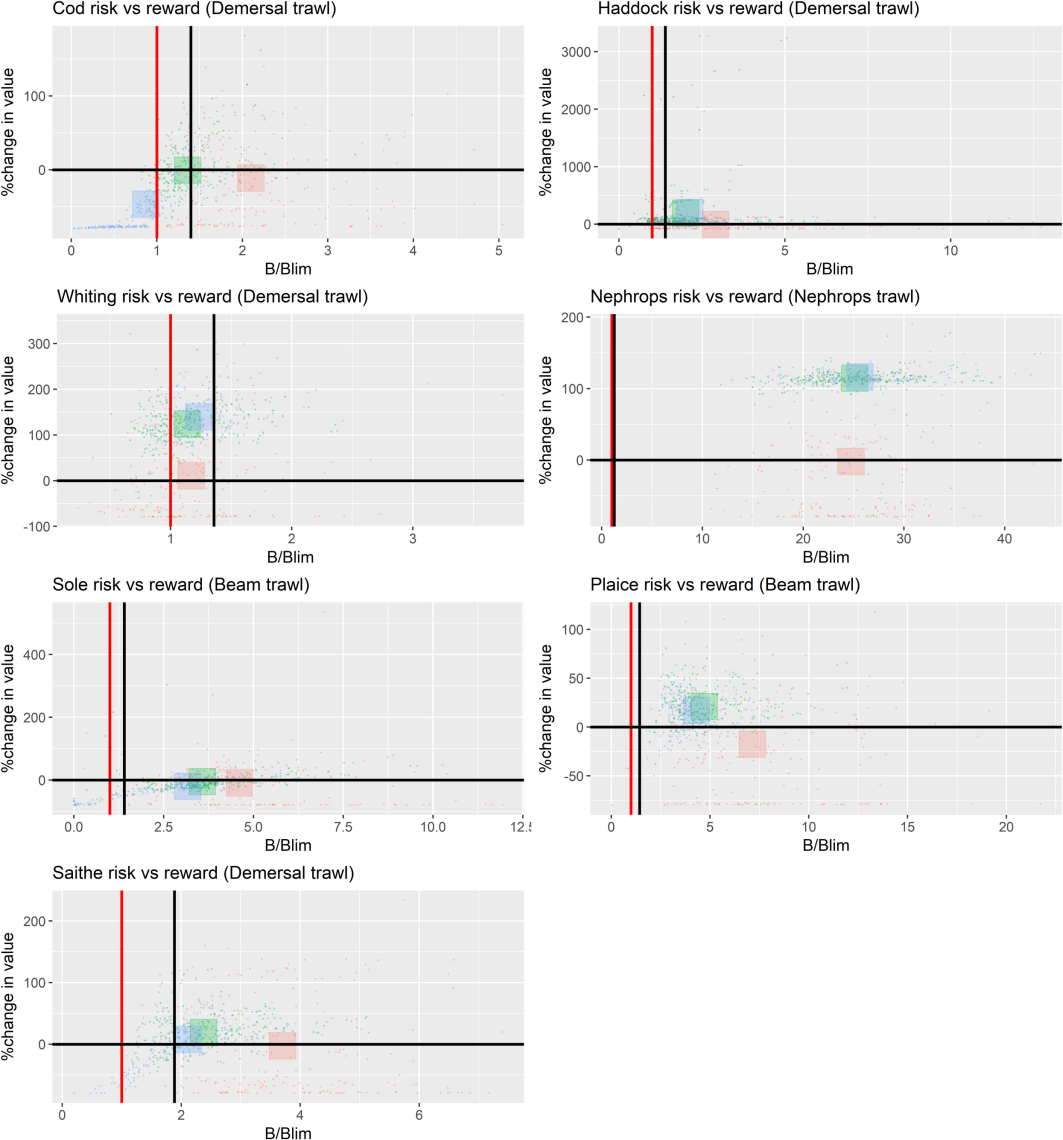
Contrast in risk-reward between 3 strategies. Strict no discards (red), discard avoidance (green), discard continues (blue). Risk by stock and reward by value to the key fleet* targeting the stock in the NSMAP scenario, fishing at F_msy_ by 2020 and safeguarded by HCR Type 4 (See Fig 4). The red vertical line in each panel shows B_lim_ and the black line in MSY B_trigger_. Each small point represents an outcome for a single model parameterisation from the full set given coloured with respect to the strategy implemented. The large squares show the mean response averaged across the parameter set and coloured with respect to the strategy implemented. *(Key fleets: cod, haddock, whiting, saithe = demersal trawl and seine; *Nephrops* = Nephrops trawls; plaice and sole = beam trawls).

**Fig 7:**
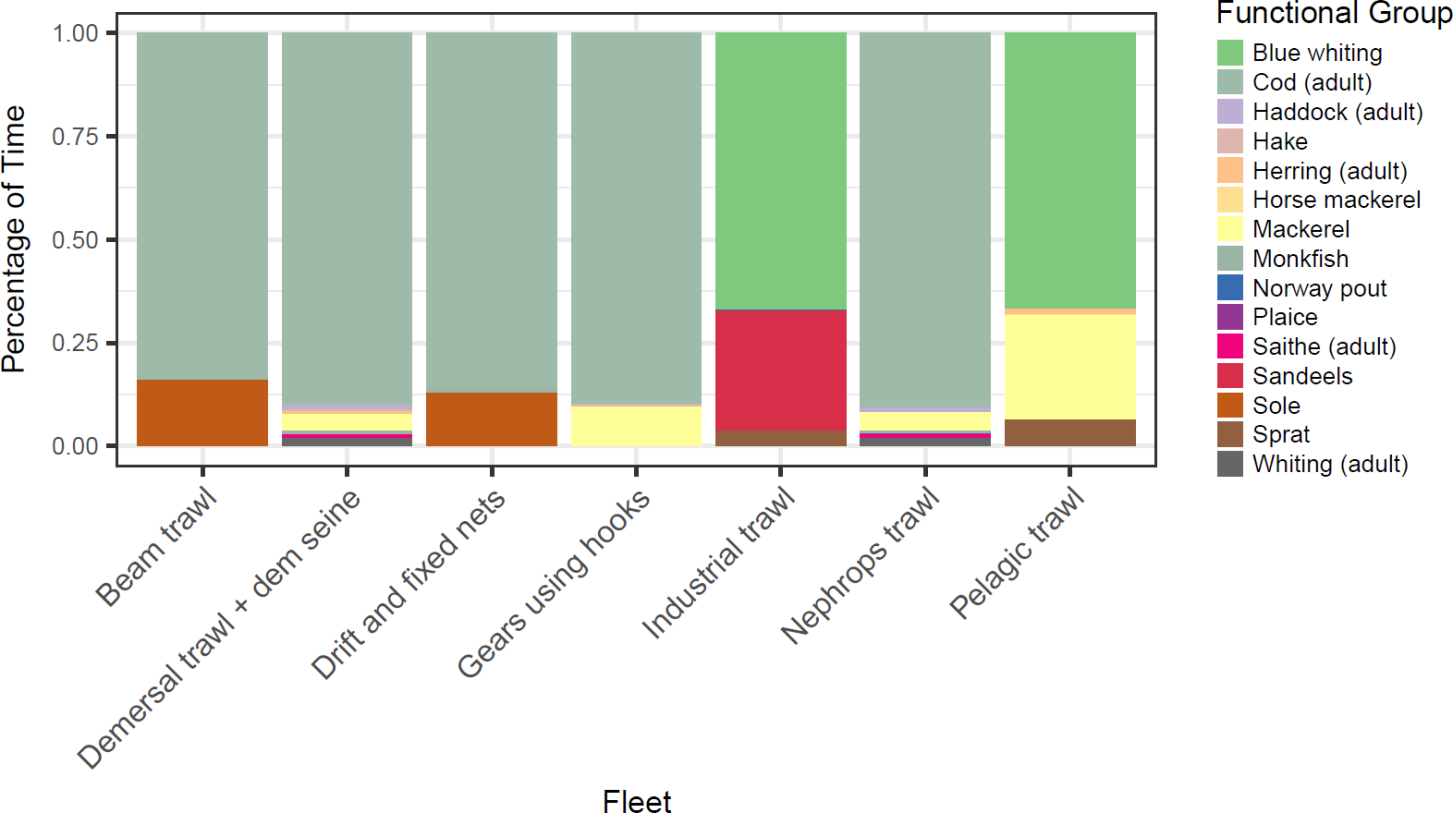
Choke species in NSMAP scenario fishing at F_msy_ by 2020 safeguarded by HCR Type 4 with strict no discards (See Fig 4). Each section within a single bar shows the percentage of years in which each stock forms the choke species (averaged across each model simulation for this single strategy).

The highest average value to the key fleet that fishes for cod (i.e. the demersal trawl and seine fleet) is achieved under the discard avoidance scenario because biomass and fishing opportunities are sustained at higher levels over the long-term (Fig 6). The strict no discarding scenario results on average in lower value because effort of the fleet becomes constrained when cod becomes a choke species (Fig 7), denying the value of other quotas to be fulfilled. Cod are also the primary choke species for many other fleets; with the exception of pelagic and industrial trawlers for which blue whiting become the primary choke species.

**Fig 8:**
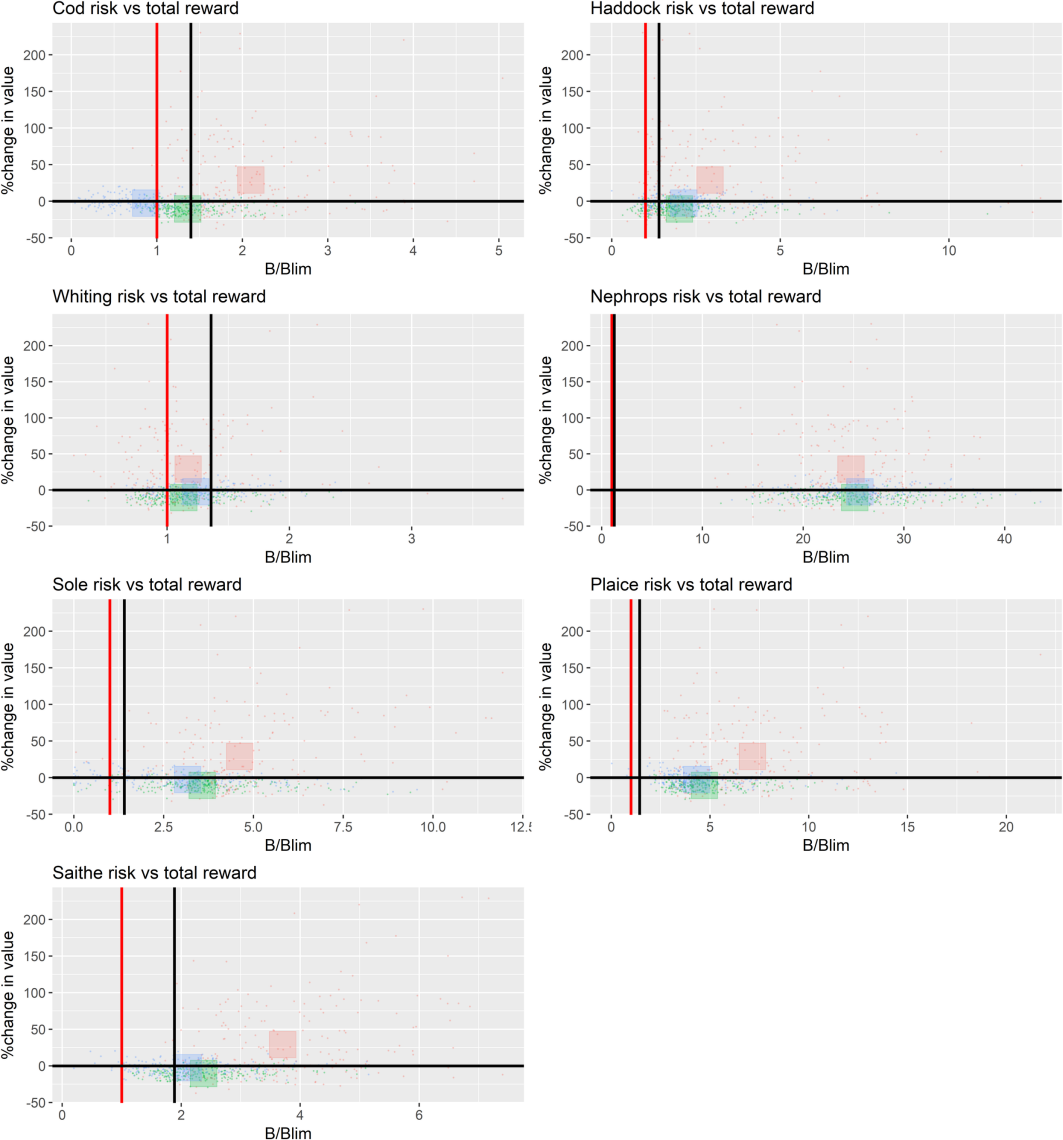
Contrast in risk by stock and total reward (combined value of the species in the NSMAP across all fleets) between 3 strategies. Strict no discards (red), discard avoidance (green), discard continues (blue). The red vertical line in each pane shows B_lim_ and the black line in MSY B_Trigger_. Each small point represents an outcome for a single parameterisation from the full set coloured by strategy implemented. The large squares show the mean response averaged across parameterisations and coloured by strategy implemented.

Under a strict no discard policy, the choke effects of cod mean that the value of *Nephrops* fisheries is reduced. In contrast, when discarding occurs, the fleet achieves its highest value, (i) because it can uptake its quota, and (ii) because the abundance of *Nephrops* is higher due to a reduction in predation on them resulting from lower cod abundance (‘predation release’). Overall however, the total value of landings by all fleets in the plan is highest under the strict no discard policy (Fig 8).

**2. Flexibility to set the F on each species within a range**

**Fig 9:**
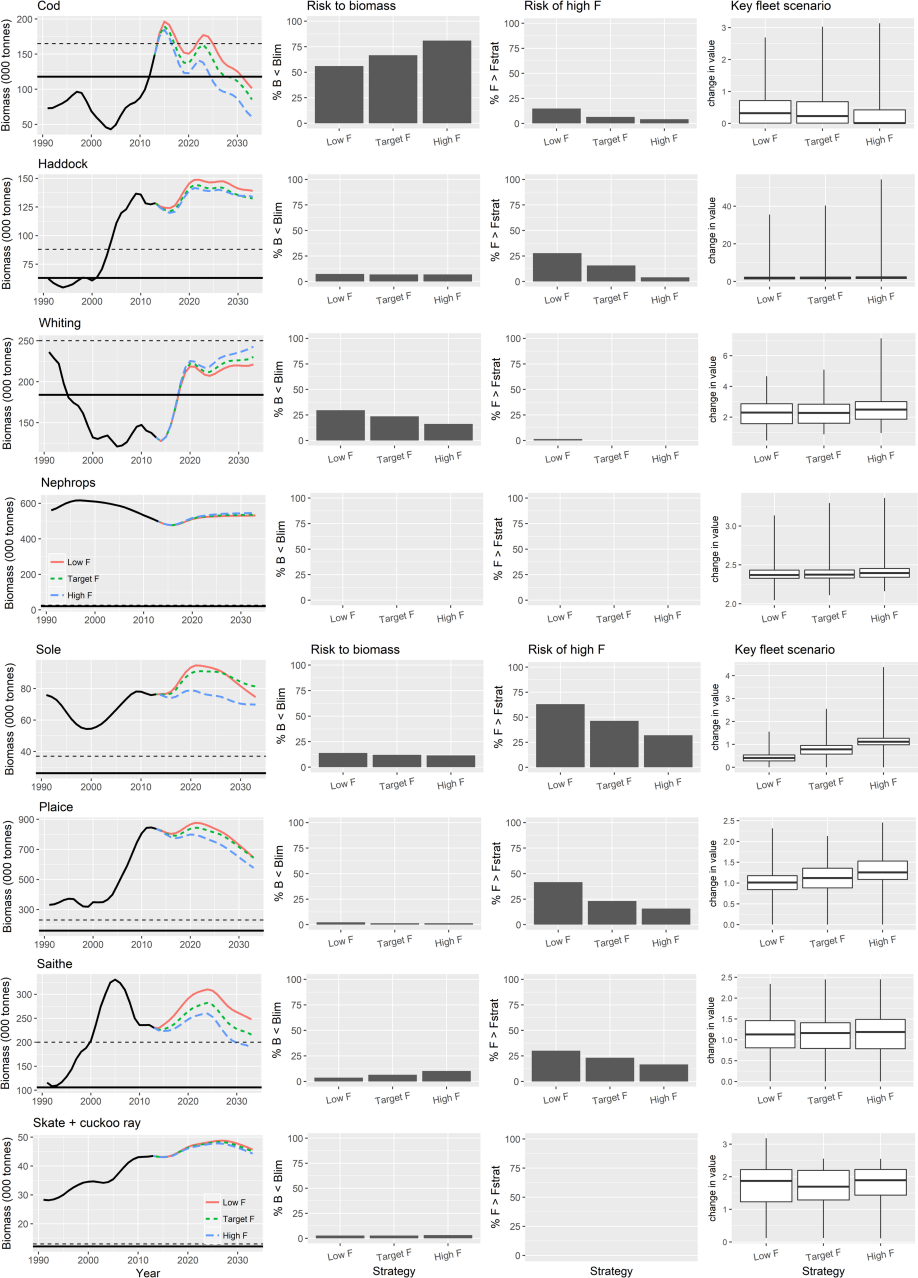
Contrast between fishing effect of ranges in fishing mortality. Low F (red), high F (green) and F_msy_ (blue) implemented with NSMAP strategies with safeguard and using realistic harvest control rule type 4 (see Fig 4) and fishing at target levels by 2020 with discarding continuing. The solid black line in the first column of plots shows B_lim_ and the dotted line MSY B_trigger_.

In comparison with the discard regulation options (Fig 5) ranges of fishing mortality have a much lesser effect on the biomass or the risk of exceeding management targets for any of the key target species in the plan. If discarding were to continue then even fishing continually at a low F scenario would result in a declining trend in biomass in the majority of species in the plan, with for example, cod falling below B_lim_ during the course of the forecast (Fig 9). The reason for this is that the realised F almost always exceed the target F when discarding continues. High F strategies consistently lead to lower long-term biomass of target species with saithe falling below MSY B_trigger_ in the forecast. In contrast, whiting and *Nephrops* attain higher biomasses through release from predation by depleted species, particularly cod.

The risk of landed F > target F is less when the target F is high because the higher level is rarely achieved. When stock biomass has a high risk of going below its reference points, long term economic performance of the fleets is reduced, lower value being obtained because quotas get reduced as fishing rates are adjusted downwards by the harvest control rule. High F strategies reinforce this, while low F strategies lead to improved long term fisheries values as stock size and quotas increase.

**3. Allowing for safeguards**

**Fig 10:**
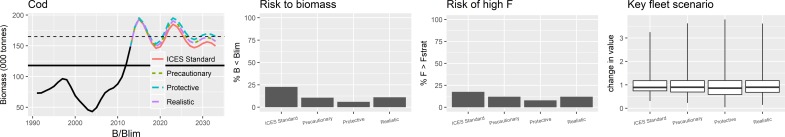
Modelled effect of HCR types on cod metrics for a scenario implementing the NSMAP with fishing at F_msy_ by 2020 and incorporating discard avoidance.

The choice of the configuration of harvest control rule types to implement safeguarding becomes of importance only when the biomass of the species is close to its reference point. As is the case for cod when fished at F_msy_ by 2020 with discard avoidance measures included so that the total available quota is always taken (Fig 10). For this scenario, only the Protective HCR (Fig 3, Type 3) on cod leads to a biomass above biomass MSY B_trigger_. The ICES standard advice rule (Fig 3, Type 1) is the least precautionary leading to the greatest risk of B < B_lim_.

**4. Time to achieve Fmsy**

Moving to implement F_msy_ sooner rather than later in general results in faster changes in stock biomass, but like F ranges and harvest control rule types, the effects are overshadowed by choices in the discard policies. In the short-term, moving to F_msy_ quickly requires the fishery to forgo some catch as evidenced by the reduction in the value generated by the fishery. In the long-term, the model converges to similar solutions.

**5. Contribute to the ecosystem-based approach to fisheries**

**Fig 11:**
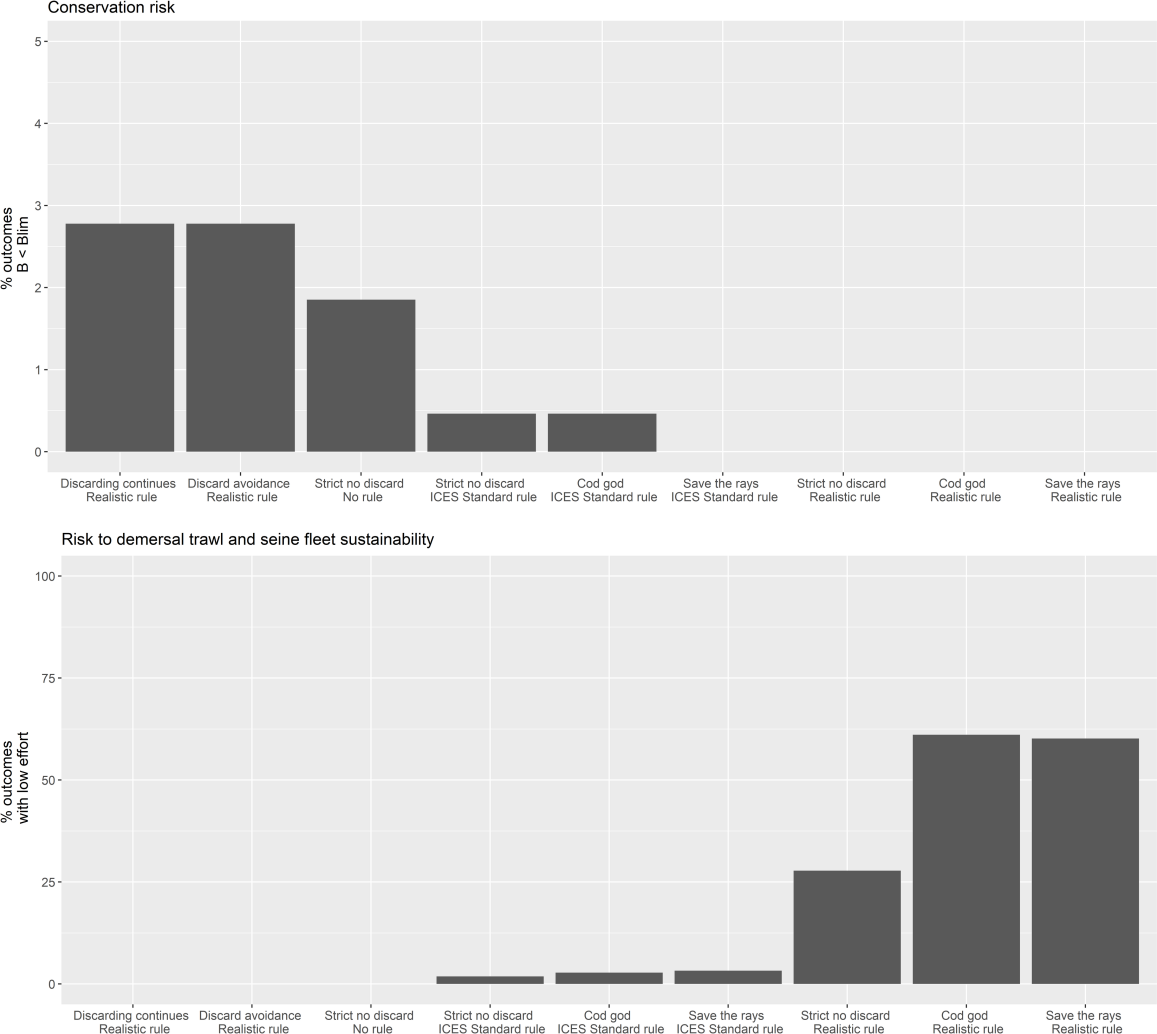
Contrast in risk to conservation species biomass (top) and sustainability of the principal fishery (bottom). Conservation species—common skate and cuckoo ray). Principal fishery—demersal trawl and seine fleet. Low effort is defined as ≤ 10% of fishing effort in 1990) between 9 strategies each fishing at F_msy_ by 2020. The contrast between bars 1, 2 and 7 shows the change in risk due to change in discard regulation options only as each is safeguarded by HCR Type 4. Bars 3 and 4 contrast the change in risk when the safeguard as applied in column 7, is either removed or altered to HCR Type 1 (See Fig 4). Bars 5 and 8 demonstrate the effect of a management strategy focused on reducing risk to the depletion of cod (‘Cod god’ strategy, see text, using HCR Type 1 and 4 respectively). Bars 6 and 8 demonstrate the effect of a management strategy focused on reducing risk to the depletion of skates and rays (‘Save the rays’ scenarios, see text, using HCR Type 1 and 4 respectively).

Fishing on commercial stocks also poses risks for the by-catch of species of conservation concern such as skates and rays. The risk that these species fall to biomass levels lower than B_lim_ was reduced when an NSMAP strict no discard strategy or a ‘Cod god’ strategy was modelled (Fig 11). Incorporating safeguards to the NSMAP no discard strategy, so that fishing effort is reduced as the biomass of target stocks falls below MSY B_trigger_, can further greatly reduce the risk to conservation species: even a simple ICES standard advice rule Type 1 can be effective and a Realistic HCR Type 4 can eliminate the risk of depletion of conservation species in the model (given our approximate model generated reference points, Table A in S1 Supporting Information). Similarly, the risks to skate and ray biomasses can be successfully managed down through a ‘Save the rays’ strategy. Little difference between the NSMAP strategies with continued discarding and discard avoidance strategies was found and both can lead to higher risk to conservation species since non-commercial species caught as by-catch may continue to be discarded unless they are explicitly defined with a zero TAC.

As the risk to conservation species is managed down so the risk that the fishery becomes unsustainable goes up (Fig 11). For example, when continued discarding and discard avoidance policies are implemented in the NSMAP strategy with a Realistic HCR or strictly no discards are allowed without an advice rule, there is little risk to the fishery. However, if the strictly no discard policy is included in the ‘Cod god’ or ‘Save the rays’ conservation strategies using a Realistic HCR, then the risk that the fishery effort reaches < 1% of 1990 values is greater (>25%) and particularly so for the conservation HCRs (>50%). A trade-off can be found between conservation and fishery sustainability if the NSMAP strictly no discard, Cod god or Save the rays strategies are coupled with an ICES standard rule (<1% risk to skates and rays and <5% risk to the fishery). In this instance, the Realistic control rule with a conservation species (cod or skates and rays) leads to a step change from fishing at F_msy_ to near zero fishing at B_lim_ with the net effect that the main fleets catching species of conservation concern are reduced to very low effort from which it can take many years for the fishery to recover. A less protective rule such as the ICES standard HCR more gradually reduces fishing effort, which although slightly risker leads typically to biomass of species above B_lim_.

**Fig 12:**
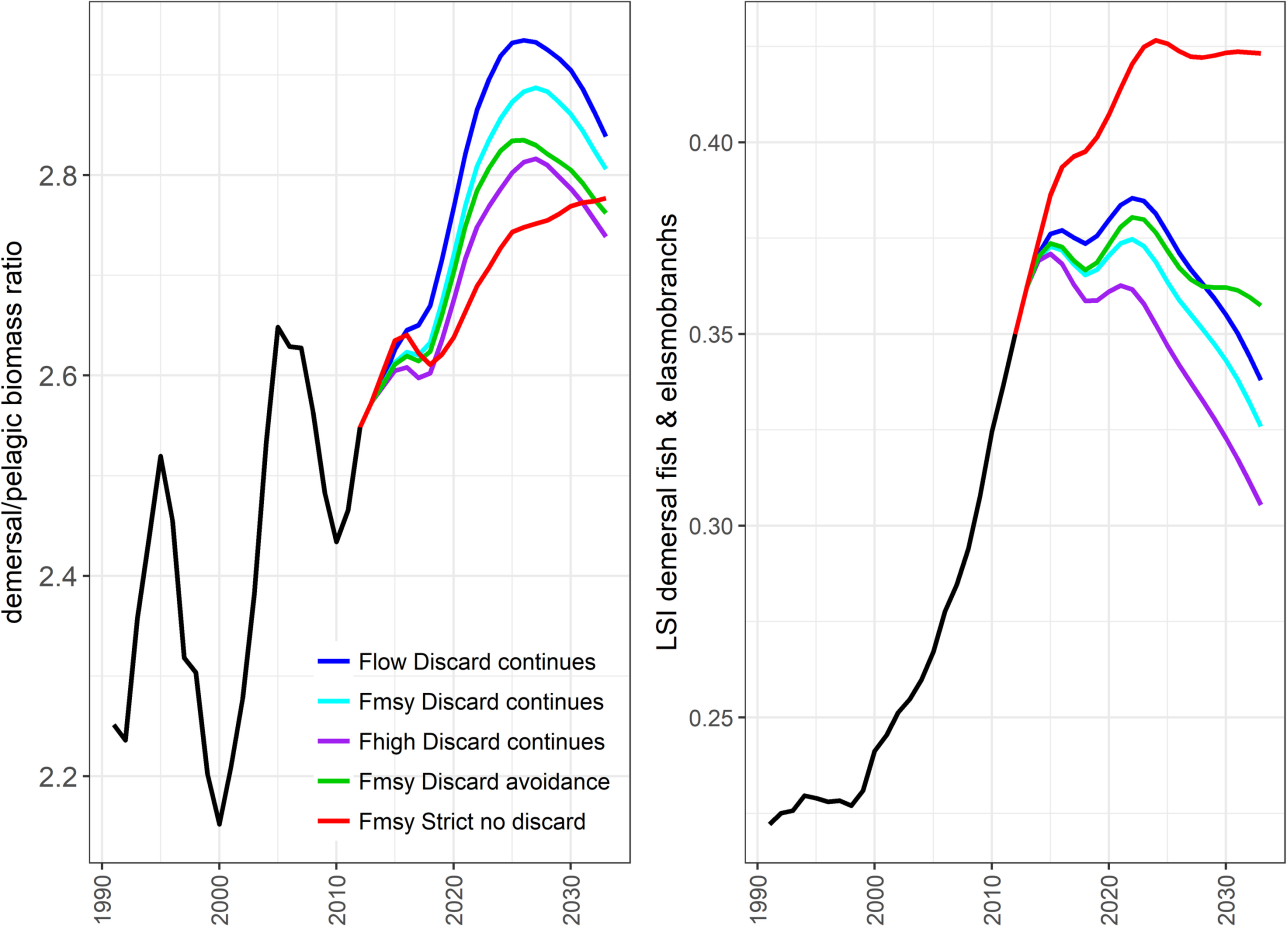
The Large Species Indicator (LSI, right) and ratio (left) of biomass of demersal species to pelagic species contrasted across multiple strategies. Strategies include all NSMAP with ICES standard advice rule Type 1 as the safeguard and constant time frame rule. Pelagic groups include sprat, herring, blue whiting, miscellaneous filter feeding fish, mackerel and horse mackerel and demersal fish include all other fish and elasmobranch groups. LSI includes only those species surveyed by the North Sea International Bottom Trawl Survey (see Lynam and Mackinson, 2015). Note all scenarios are equal for the period 1991–2012.

**Fig 13:**
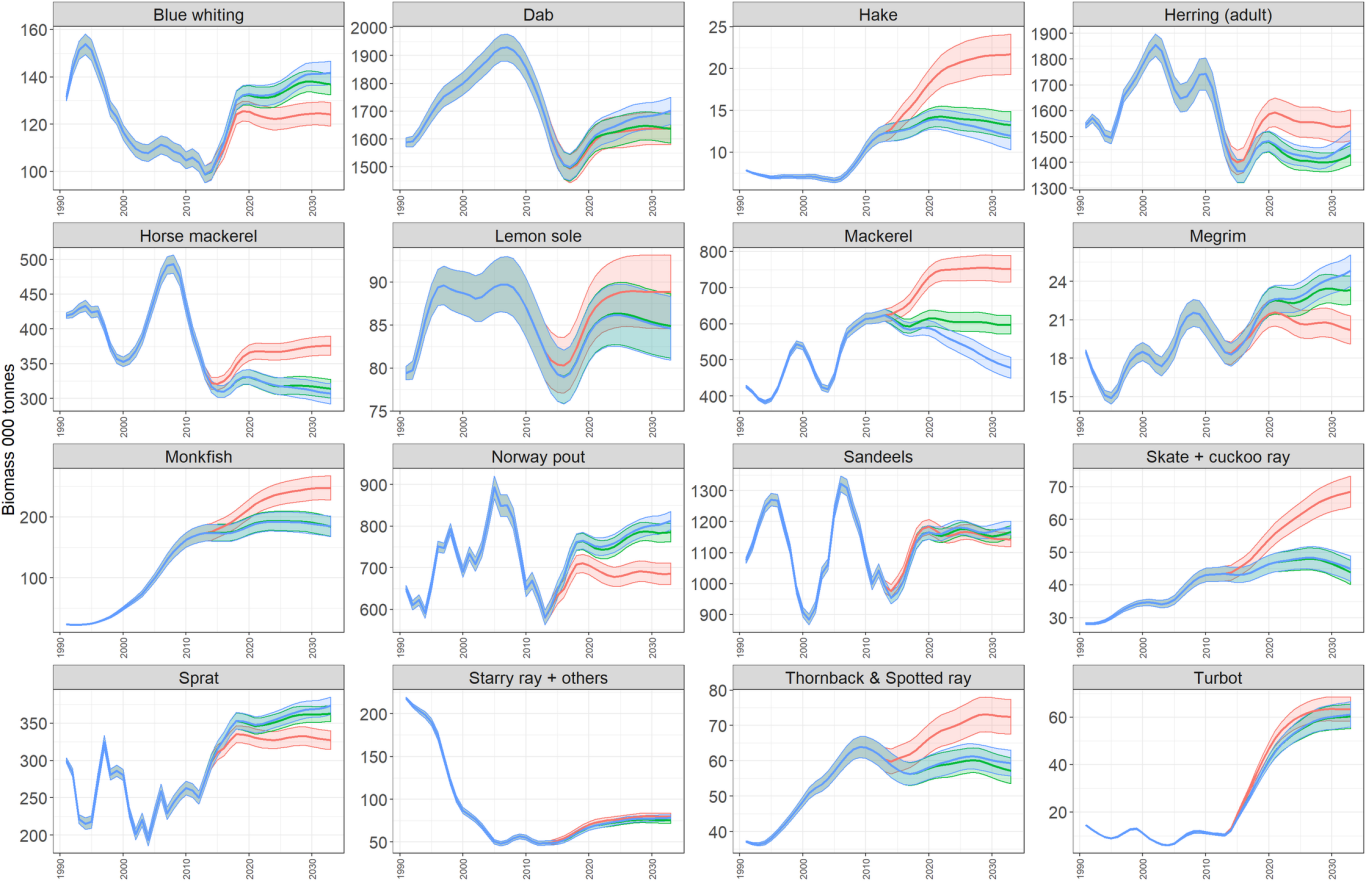
Biomass trajectories of additional species of interest. Shows the contrast in outcomes between 3 strategies, strict no discards (red); discard avoidance (green) and discard continues (blue) with 95% confidence interval.

**Fig 14:**
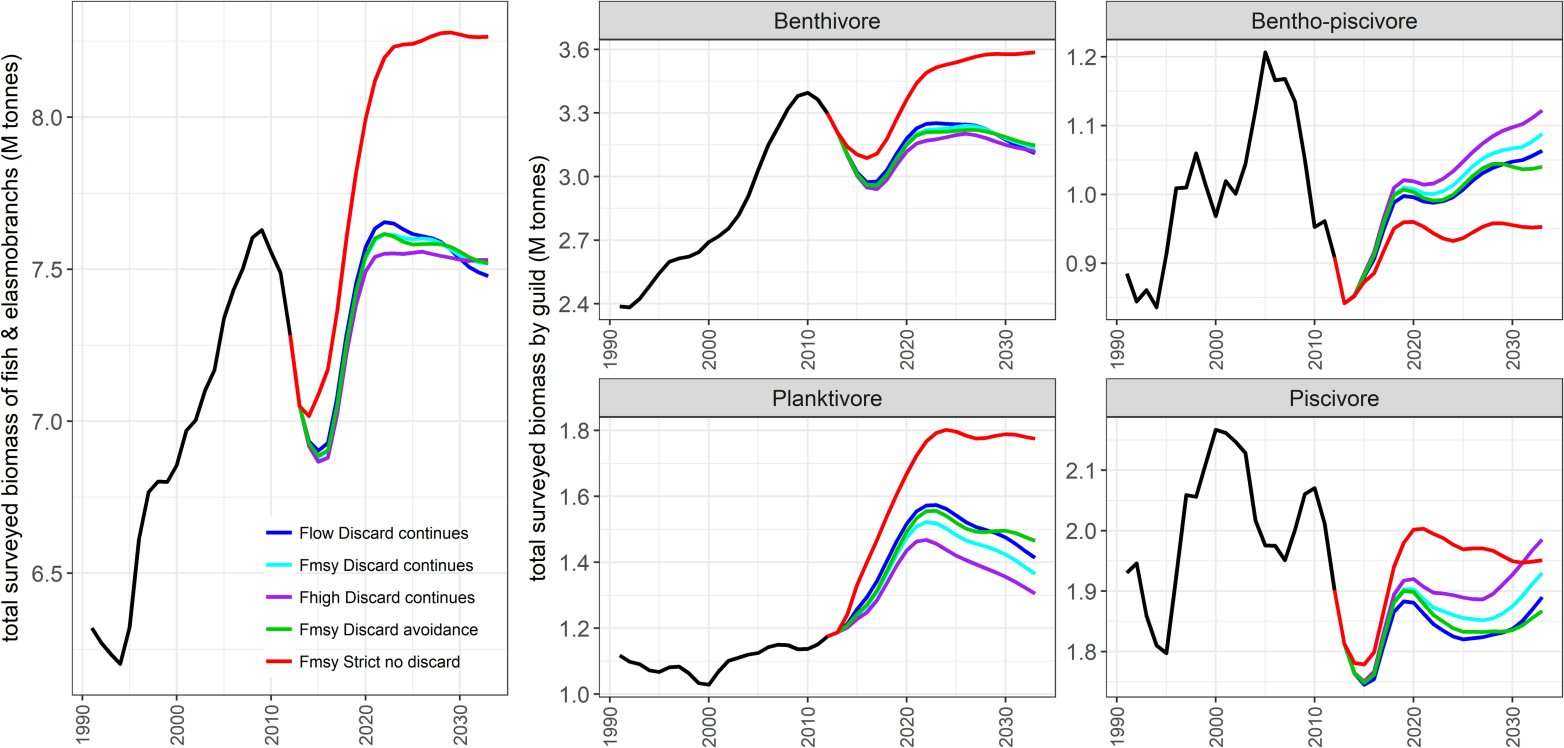
Foodweb indicators contrasted across multiple strategies. All NSMAP strategies with ICES standard advice rule Type 1 as the safeguard and constant time frame rule. Left—total biomass of fish and elasmobranchs and right—biomass by trophic guild, including only those species surveyed by the North Sea International Bottom Trawl Survey (see Lynam and Mackinson, 2015). Note all scenarios are equal for the period 1991–2012.

**Fig 15:**
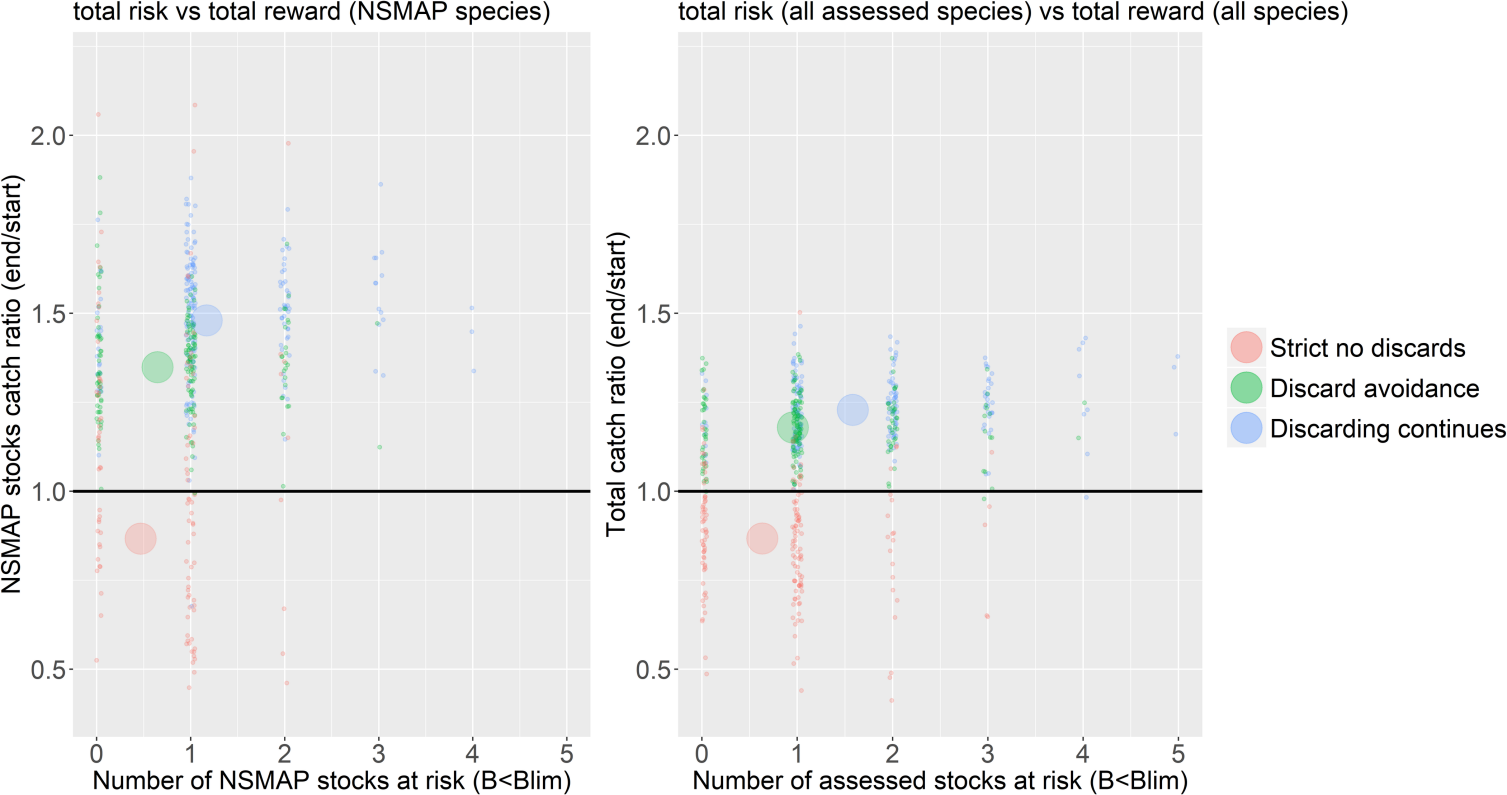
Contrast in risk of depletion of stocks against the reward of total landed catch. Risk is the number of stock below their individual B_lim_ reference points. The vertical axis shows the ratio of total tonnage caught at the end of the forecast, i.e. the average of the last five years, to the initial catch at the start of the forecast period. The pane of the left is specific to the stocks in the NSMAP, whereas the pane on the right includes all species with a reference level in the estimate of risk and all fish and elasmobranch stocks caught by fisheries in the catch ratio. The risks and rewards are contrasted between 3 strategies; strict no discards (red), discard avoidance (green), discard continues (blue). The black horizontal line shows the level at which the tonnage caught at the end of the simulation is equal to the start, points above this line represent runs with increased tonnage caught. Each small point represents an outcome for a single parameterisation from the full set coloured by strategy implemented. The large circles show the mean response averaged across parameterisations and coloured by strategy implemented.

Recent management has contributed to an increase in biomass of demersal species such that the ratio of demersal to pelagic species biomass in the ecosystem has increased (Fig 12, panel (a), black line). Interestingly, the effect of a strict no discards scenario appears to be greater for pelagic species (including herring, mackerel and horse mackerel), the proportion of demersal species relative to pelagic species increasing less than when discarding continues. Despite this, the proportion of large bodied demersal fish species (cod, haddock, plaice, saithe, monkfish, spurdog, hake, catfish (wolf-fish), turbot, brill, thornback and spotted ray) is greatest under a strict no discards scenario (Fig 12B) since this indicator is driven strongly by the biomass of the large piscivorous species cod. Among the additional species of interest, sprat, blue whiting and megrim are all depressed when discards are strictly forbidden, while hake, herring, Horse mackerel, mackerel, monkfish, skate and Cuckoo ray, Thornback and spotted ray all increase in biomass (Fig 13). Possible changes in the community structure of the North Sea reveal again the impact of the strict no discard scenario (Fig 14). The biomasses of most trophic guilds increase under the no discard scenario, leading to an increase in biomass overall, but a relative decrease in bentho-piscivores (dominated by Norway pout an important prey species for larger piscivores). Finally, a comparison of the risks and reward to species in the proposed NSMAP versus a broader ecosystem perspective that considers impacts on all assessed species, shows similar scale impacts and that while discard avoidance reduced the risk to the number of stock where B<Blim, they maintain a positive reward to long term catches (Fig 15).

## Discussion

Fully implemented, the Basic Regulation of the CFP means it is illegal (under the Landing Obligation) to discard any catches in excess of quota, so fishing vessels would have to stop fishing early in the year once their quota for the most limiting stock becomes exhausted. However, various mechanisms serve to soften this hard implementation and allow them continue fishing such as *de minimis* and high survivability exemptions, inter-species quota flexibility and quota swapping. There is also evidence that the discard ban may be difficult to enforce [30, 31]. Our comparison between the strict no discard and discard avoidance modelled strategies represent different degrees of implementation or fleet response to the landing obligation, or the effects of its staged implementation from 2016 to 2019.

Overwhelmingly, results of our evaluation of the contrasting impacts of 5 key dimensions of the proposed NSMAP show that the choice of discard implementation option has the largest effect on the risk to commercial species biomass and reward to fisheries. Flexibility to set ranges in F and the way in which safeguards are applied have limited impact, being most effective at times when stock biomass is close to conservation reference values. Although some yield to demersal fisheries would be lost in the short-term, results also indicate that a strict policy on stopping the discarding of over-quota fish should lead to further recovery of exploited stocks and sustainable fisheries in the long-term, with the total value landed by all fisheries combined increasing (Fig 8) despite decreases in total tonnage caught (Fig 15). Positive benefits are also seen to occur in the discard avoidance strategies, which perhaps reflect more closely the reality of discard policy implementation given the various mechanisms that aim to keep the fleet operational while minimising discards. In particular, results indicate that the risk of stock conservation limits being compromised is typically lower than when discarding continues, while the fisheries value—and thus their sustainability—is always higher than under strict no discards, and often higher or on a par with continued discard scenarios (Figs 5, 6 and 15).

Importantly, the risk of depletion of species of conservation concern (skates and rays) is also diminished when the strict no discard option is implemented, leading to a win-win for commercial and conservation species. Model simulations suggest that the risk can be eliminated by implementing Realistic safeguards through a harvest control rule (Type 4), which is more conservative than the ICES standard advice rule (Type 1). Mirroring the risk to stock sustainability, conservation strategies for protecting above all others cod or skates and rays poses an obvious risk to the sustainability of dependent fisheries, which is intensified when precautionary harvest control rules are applied (Fig 11). In this case the ICES standard HCR is more beneficial to the sustainability of fisheries. This contrast illuminates the stock-fishery sustainability trade-offs.

Even in the strategy most likely to reflect implementation of the NSMAP (i.e where discards are avoided but not wholly eliminated) as the model ecosystem adjusts to a new equilibrium, the indirect food-web effects arising from changes in demersal predators result in declines in some prey species. Particularly bentho-piscivorous species such as Norway pout, some planktivores such as Blue whiting and sprat, and some smaller bodied demersal piscivores such as megrim (Figs 13 and 14). Such changes in the food-web structure are an inevitable consequence of objectives to sustain important commercial fish stocks and their fisheries.

The implementation of the “Avoiding Discarding’ option gives quite a generous appraisal of the fleets ability to selectively target species at lower risk. Modelling fleet behaviour is notoriously difficult due the vast array of regulatory and human decision-making process that affect the outcome, and are thus subsumed as implementation errors. Here we have made a transparent, albeit simplified representation of the mechanism for avoiding discards, and have chosen not to set implementation error a priori, but to allow it to emerge from the interaction among the HCRs and regulatory conditions (see section 3.6 of supplementary material). There are some slight inconsistencies in the proportions of catch discarded for each regulation (see section 3.6 of supplementary material), however since the behavioural response to each regulation has a far more significant effect on the results and there is a clear order of ranking between results from each regulation, this does not affect our conclusions.

The management strategy evaluation approach applied here is state-of-the-art in screening the possible risks associated with alternative policy options, despite some controversy and misunderstanding on its use and utility [15, 32, 33]. As a tool best suited for comparing and ranking the performance of strategies it is not intended for making specific short-term predictions about stock-specific biological and fishery outcomes. More detailed models incorporating more structure in the biological dynamics of stocks and behavioural dynamics of fleets are required for that. For example, while changes in the maximum range of F values have a small impact on the overall risk and rewards relative discard policy, our analysis does not explore the myriad of possible combinations of F within the ranges among species, and how these might be adjusted from year to year in order to resolve specific problems such as reducing fishing pressure on one species or avoiding another being a choke to the fishery (see point 12 and 13 in the proposed NSMAP). In such cases, optimisation procedures with clearly defined objective functions are required to search for options with desirable outcomes. Since the way a fleet is able to respond to a discard policy has an important impact, being able to make good predictions of fleet behaviour is important for models. Tools such as FLBEIA (among others) are being constructed specifically with this purpose in mind (see [34] and references therein).

The NSMAP presents biological reference points generated by single-species methods, despite the scientific consensus that these would not be simultaneously achievable due to biological interactions among species and changes in the environment affecting productivity [35, 5]. While other reference points determined from multi-species models in the North Sea such as SMS, LeMans and EwE (e.g. [36]) could be evaluated along with the single species reference points, that comparison would be about testing alternative ‘interpretations’ of reference points and not the evaluation of the NSMAP as it stands. Nonetheless, it would make for a relevant and interesting future comparison given that the move toward multi-annual plans has implications for the increased use (and utility of) multi-species models in advice.

The scale of the modelled impacts in the North Sea EwE key run model are sensitive to specification of P/B parameters [26]. However in this analysis the P/B parameters, along with all the other Ecopath and Ecosim parameters are sampled from distributions so that the robustness of the strategies to uncertainties in the model can be measured. Of the 1000 parameterisations of the North Sea model evaluated, around 25% passed the criteria for acceptable dynamic behaviour when the alternative policies were applied. One of the features that emerges from letting the parameters ‘run free’ is the occurrence of adult-juvenile oscillatory dynamics, which occurs in the future projections. Parameter combinations that led to unstable oscillatory behaviours are the main reason that approximately 80% were screened out as having unacceptable trajectories. It should also be noted that the model simulations should not be treated as reliable future forecasts of stocks because they do not incorporate possible scenarios for how changes in the environment may affect biological process of individual stocks and the resulting cascade through the food web [37, 38].

Recognition of these technical shortcomings does not negate the findings of the MSE analysis which illuminates the relative impacts of the 5 management dimension of the proposed NSMAP. The results imply that when defining and implementing the detail of the North Sea a multi-annual plan, effort expended on getting the discard regulation options right will lead to greater measurable effects for stocks, fisheries and other environmental objectives than fine tuning targets for fishing mortality and the way in which they are adjusted.

Using a multi-species multi-fleet model allowed us to take account of the species and fleet interactions, even if it was not possible to tease apart the role that these interactions may play in every result. They have however, shown clearly that changes observed by fishermen can be captured, such as the predation effect of cod on *Nephrops* having a bearing on the stock and performance of fisheries that depend upon it [39]. It also enabled us to present information relevant to assessing the ecological and fishery trade-offs that might arise from implementation of the plan by revealing the impacts on demersal and pelagic assemblages and different fleets. In doing so we’re taking one-step closer to being able to provide scientific evidence to support ICES integrated advice approach founded upon the ecosystem approach to fisheries. The important message is that because fish and fisheries do not exist in isolation, neither should plans for their management. Any future multi-annual plans (e.g. for pelagic stocks) should be evaluated in terms of how consistent they are with this one for demersal stocks.

## Supporting information

S1 Supporting Information Technical methods of the uncertainty and MSE routine. (DOCX)
