# Supplementary material for: Evaluating the fishery and ecological consequences of the proposed North Sea multi-annual plan
Source: PLoS One. 2018 Jan 2;13(1):e0190015. doi: 10.1371/journal.pone.0190015 (PMC5749736; doi:10.1371/journal.pone.0190015)
Supplement: S1 Supporting Information — (DOCX) [file pone.0190015.s001.docx]

# Supporting information.

# Technical methods of the uncertainty and MSE routine

## Overview

The Ecopath with Ecosim (EwE) plug-in has two main stages of execution (Fig 1). Stage 1: Create possible EwE Models – uses user-specified probability distributions to sample the input parameters of which an EwE model is comprised, and saves them to disk. Stage 2: Evaluate Management Strategies (Fig 2) gets input from the user about Harvest Control Rules, Discards Policies, Quota shares for each fleet, Number of Years to Project, Observation and Implementation Errors, Maximum fractional increase in effort and Biomass limits. Applying these settings, Ecosim is then run for all selected combinations of strategies and models created in stage 1, and providing the results are plausible, they are saved to disk.

## Creating alternative ecosystem models

The process of creating alternative Ecopath models involves sampling the basic input parameters for biomass, biomass accumulation, ecotrophic efficiency (EE), production/biomass (P/B) and consumption/ biomass (Q/B) parameters using a truncated normal distribution. In addition, the diet proportions for each predator are sampled using a Dirichlet distribution. Together the sampled Ecopath and diet proportions are checked to see that they meet the criteria for EE<1, positive respiration and P/Q<0.5. The sampling process is repeated until a valid model is determined.

For each of the Ecopath models created, a set of Ecosim parameters comprising of density dependent catchability, feeding time adjustment rate, max relative feeding time, fraction of other mortality sensitive to changes in feeding time, predator effect on feeding time, QBMax/QBo and switching power are generated by sampling either a uniform or triangular distribution. Discard survival proportions, or more explicitly, the proportions of discards that survive are sampled for each fleet species combination using a beta distribution. The vulnerability parameters are sampled for all possible interactions, using the formula (Gaichas et al. 2012):

| $v_{rk}=1+e^{9(U-0.5)}$ | Equation 1 |
| --- | --- |

Where $v_{rk}$ is the vulnerability, r is the predator, k is the prey and $U$ is a value sampled from a standard uniform distribution.

A single set of all the Ecopath and associated Ecosim parameters are combined to form a single EwE model. Multiple sets combined form an approximate representation of the probability distribution for all possible EwE parameterisations. By running strategies (see next section across all these models, strategies are tested for robustness to parameter uncertainty.

## Evaluating the management strategies

Strategies are evaluated by running each one across the entire range of possible ecosystem models to produce a single outcome for each strategy-model pair. If a model produces a biomass trajectory beyond a plausible range for any strategy the model is excluded from the analysis.

The management loop, core to the evaluation, becomes active as soon as the model projects into the future, the methodological steps of which are described below.

### **Estimate a biomass from scientific surveys – the simulated ‘Survey Biomass’**

At the beginning of the year the average biomass predicted by the model over the previous 12 months is calculated. Observation error sampled from a log-normal distribution parametrised using a user specified coefficient of variation is then added to it, giving an estimate of biomass at the beginning of the year. This is the simulated ‘Survey Biomass’ which is used by the stock assessment (see section 3.2).

### **Performing stock assessment**

The results of complex assessments (which are not practical to simulate for multiple functional groups in the Ecosim MSE framework) are simulated by approximating the results of stock assessments as a weighted average of the predicted biomass and biomass estimated from surveys (Walters 2004, equations 1-6):

| $B_{t\vert t}=B_{t\vert t-1}+K_{t}^{*}(B_{t}^{*}-B_{t\vert t-1})$ | Equation 2 |
| --- | --- |

Where $B_{t|t}$ is the best (minimum variance) estimate of $B_{t}$ given all of the data up to time t, $B_{t|t-1}$ is the best prediction of the biomass at time t given only the data up to time t -1, $K_{t}^{*}$ is the Kalman gain and $B_{t}^{*}$ is the simulated “Survey” biomass at time t. The prediction ($B_{t|t-1})$ comes from Schnute’s generalization of the original Deriso model (Schnute, J. 1985), with a simplified representation of biomass dynamics:

| $B_{t\vert t-1}={gB}_{t-1}e^{-FishMort-NatMort}+wR_{t}$ | Equation 3 |
| --- | --- |
| $g=\max_{} \{0,e^{\left( \frac{Ecopath\_BA}{Ecopath\_B} \right)}-\mathrm{RecruitOverPop}\}$ | Equation 4 |

Where g is the growth rate, Ecopath_BA is Ecopath biomass accumulation, Ecopath_B is the Ecopath Biomass, **RecruitOverPop** is the percentage of total population that will recruit, and $wR_{t}$ is the biomass recruited estimated using the Beverton-Holt Stock Recruit model:

| $wR_{t}= \frac{\alpha\times S_{t-1}}{\beta+S_{t-1}}$ | Equation 5 |
| --- | --- |
| $\alpha=RecruitOverPop\times Start Biomass\times RatioBt$ | Equation 6 |
| $\beta= RatioBt\times Start Biomass$ | Equation 7 |
| $S_{t-1}=B_{t-1}e^{-Z_{t-1}}$ | Equation 8 |

Where **RatioBt** is the Ratio of biomass at time to Ecopath base biomass for 50% of recruitment and **RecruitCV** is the coefficient of variation of the actual recruits around the predicted recruits from the stock recruit model.

The Kalman gain is the proportion that the predicted biomass variance (Var(B_t|t-1_) is of the total predicted and observed biomass variances:

| $Var(B_{t\vert t-1})=\frac{{(RecruitOverPop\times RecruitCV)}^{2}}{1-g^{2}}$ | Equation 9 |
| --- | --- |
| $K_{t}^{*}=\frac{Var(B_{t\vert t-1})}{Var(B_{t\vert t-1})+Var(B_{t}^{*})}$ | Equation 10 |

### **Determine the Quota using the HCR**

A quota is determined for each functional group that has a HCR using:

| $F_{i}=\left\{ \begin{matrix} 0 \\ (B_{i,t\vert t}-B_{i}^{min})/(B_{i}^{max}-B_{i}^{min})\times F_{i}^{max} \\ F_{i}^{max} \end{matrix} \right. \begin{matrix} B_{i,t\vert t}<B_{i}^{min} \\ B_{i}^{min}<B_{i,t\vert t}<B_{i}^{max} \\ B_{i}^{max}<B_{i,t\vert t} \end{matrix}$ | Equation 11 |
| --- | --- |

Where $F_{i}$ is the instantaneous landings mortality rate, $B_{i,t|t}$ is the stock assessment estimate of the biomass for species *i,* $B_{i}^{min}$ is the biomass at which the F from the HCR declines to zero and $B_{i}^{max}$ is the Biomass at which the HCR F arrives at $F_{i}^{max}$, the maximum F, which is in many cases MSY F.

This $F_{i}$is then converted into quotas for each fleet-species combination using:

| $Q_{i,j}^{*}=F_{i}B_{i,t\vert t}q_{i,j}$ | Equation 12 |
| --- | --- |

Where $q_{i,j}$ is the share of the quota of species *i* assigned to fleet *j*.

HCRs can be specified as either Target HCRs or Conservation HCRs:

- *Target HCRs –* normal HCRs, used as above to determine an F given the biomass of a functional group.
- *Conservation HCRs* - calculates an upper limit for the Target HCR F, so that if it is above Conservation HCR F the Conservation HCR F will be used instead.

Both Target and Conservation HCRs can be specified as a traditional HCR (S1 Fig) or as a Multi-level HCRs (S2 Fig) which allow a greater degree of flexibility in the HCR shape by enabling the specification of a minimum fishing mortality as well as a biomass level known in the plugin as the “Biomass step” at which the F steps down to the minimum fishing mortality.

~~
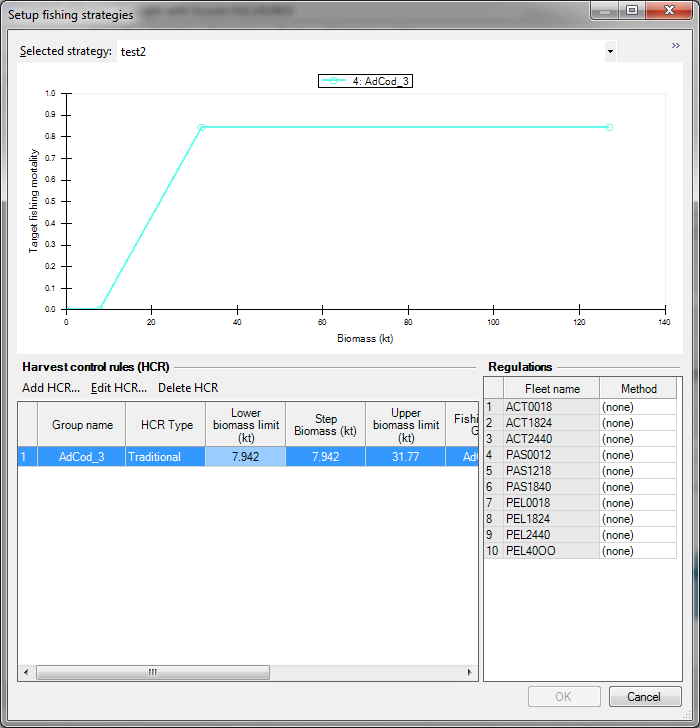
~~

**S1 Fig . Screenshot of a traditional HCR plotted by the MSE Plugin**

~~
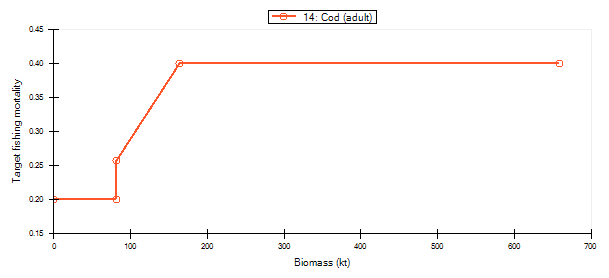
~~

**S2 Fig. Screenshot of a Multi-level HCR plotted by the MSE Plugin.**

### **Determine maximum effort for the year**

The increase in effort is limited between years to represent realistic limits in the rate of growth of fishing capacity. As a consequence the maximum effort for a given year is calculated as follows:

| $l_{j}^{max}=l_{j}^{prev}+l_{j}^{prev}\times\kappa_{j}$ | Equation 13 |
| --- | --- |

Where $l_{j}^{max}$ is the maximum effort for fleet *j*, $l_{j}^{prev}$is the effort at last time step of the previous year and $\kappa_{j}$ is the maximum percentage increase in the effort between years.

### **Determine relative effort and discard rates according to regulations**

Regulations are settings that can be applied uniquely to each fleet and specify whether the fleet is subject to a discard ban or not. They are also a way of specifying how the fleet might behave in response to a discard ban, that is, whether or not they are able to avoid species to prevent them becoming choke species.

Accurately modelling fleet behaviour is extremely difficult. To overcome this problem two regulation methods were specified, the ‘Weakest stock’ and ‘Selective Fishing’, both of which represent different degrees of implementation of (or fleet response to) a no discard policy. The ‘Weakest stock’ represents the possibility that fleets are incapable of being selective of its catch composition while ‘Selective Fishing’ represents the possibility that fleets are 100% capable of selecting their catch composition. While both scenarios are unlikely to be true, it is a fair assumption that the reality is somewhere between these two possibilities. Consequently the results from these two regulations should be considered together as a range of what is possible given a no discard policy.

Three first 3 of the 5 options are applied in this analysis.

#### 3.5.1 ‘Weakest stock’ (=Strict no discarding)

The weakest stock regulation represents a no-discards policy. The effort required to catch 1/12^th^ of the quota of each species is calculated each month (each time step) and then set equal to the minimum effort value calculated (the effort required to catch the ‘weakest stock’), to ensure that none of the quotas will be exceeded throughout a year. The weakest stock is calculated each month and in some cases may change over the course of a year. This is due to fluctuations in the biomass of each species, causing the effort required to catch its quota to vary; as the biomass of a species increases or decreases, catching it becomes easier or harder respectively and therefore the effort required decreases or increases respectively.

The fleet has no ability to alter its catch composition (i.e. unselective), meaning that the species that requires the least effort to fulfil its quota becomes the bottleneck or ‘choke species’ and determines the amount of effort deployed. Note that species that are defined as non-target species without a quota are exempt from being a ‘choke species’ and can be discarded. Presently, discards are allowed for fish below minimum size if they have been specified in the basic Ecopath model.

Step-by-step explanation

For each fleet that has a quota for at least one functional group, at each time step:

1. Cycle through each functional group for which the fleet has a quota and calculate the effort required to catch the 1/12^th^ of the functional group's yearly quota using:

| $E_{j}^{T}=\frac{q_{i,j}q_{i}^{T}}{M_{i}F_{i,j}B_{i}}$ | Equation 14 |
| --- | --- |

Where $E_{j}^{T}$ is target effort, $q_{i,j}$ is the quota share, $q_{i}^{T}$is 1/12^th^ of the yearly target quota, $M_{i}$ is the density dependent catchability multiplier at current timestep, $F_{i,j}$ is the fishing mortality, $B_{i}$ is the biomass.

1. Repeat step 1 with any conservation HCR Fs using:

| $E_{j}^{C}=\frac{q_{i,j}q_{i}^{C}}{M_{i}F_{i,j}B_{i}}$ | Equation 15 |
| --- | --- |

Where $E_{j}^{C}$ is the conservation effort, $q_{i}^{C}$ is the conservation quota.

1. If the target effort is less than the conservation effort use the target effort, otherwise use the conservation effort.
2. From all the efforts calculated for a fleet, apply the lowest to the current time-step. By applying the lowest we ensure that the main objective of this regulation is achieved, to never catch beyond the quota of any functional group.
3. If the effort is outside the upper effort limit reset the effort to the upper limit (see section Determine relative effort limits for the year)
4. Set the proportions landed and discarded to the proportion in the EwE model
5. Set the proportion surviving being discarded to the survivability value sampled for the current model

#### 3.5.2 ‘Highest value’ (=Continued discarding)

The highest value regulation is a discard permitting policy that assumes that the fleet will catch the entirety of the highest valued quota. The highest value quota in the fleets’ portfolio is calculated at the beginning of each year based on quota and price. The effort required to catch 1/12 of the highest values quota is calculated each month. Any fish caught beyond quota for other stocks are discarded.

Step-by-step explanation

For each fleet that has a quota for at least one functional group:

1. At the beginning of each year determine which functional group has the highest value quota (where value = price x quota)
2. For each timestep
   1. Calculate the effort that would land the quota

| $E_{i,j}^{T}=\frac{q_{i,j}q_{i}^{T}}{m_{i,j}^{l}M_{i}F_{i,j}B_{i}}$ | Equation 16 |
| --- | --- |

Where $E_{i,j}^{T}$ is the effort to catch the target quota and $m_{i,j}^{l}$ is the proportion of the catch that is landed and i is the index of the highest value group.

- 1. Calculate the effort required to catch the conservation quota for this highest value group if a conservation quota exists:

| $E_{i,j}^{C}=\frac{q_{i,j}q_{i}^{C}}{m_{i,j}^{l}M_{i}F_{i,j}B_{i}}$ | Equation 17 |
| --- | --- |

- 1. Let $E_{j}=\min_{} \left( E_{i,j}^{T}, E_{i,j}^{C} \right)$
  2. If the effort is outside the upper and lower limits reset the effort to the breached limit (see section *Determine relative effort limits for the year*)
  3. For each functional group for which the fleet has a quota:
     1. Estimate the catch if the fleet was to fish at the current level for 12 months

| $C_{i,j}=E_{j}M_{i}F_{i,j}B_{i}$ | Equation 18 |
| --- | --- |

- - 1. if the Catch is greater than the smallest quota from either the conservation and target HCR/prop landed as set in EwE then
       1. Set the proportion landed to quota/catch
       2. Set the proportion surviving being discarded to the value sampled for the current model
    2. If the catch is not greater than the smallest quota
       1. Set the proportion landed to the proportion in the EwE model

Note that there are some inaccuracies in the proportions of the catch discarded. These inaccuracies are rooted in the problem of combining discard proportions specified in the EwE model and the discard proportions calculated by the plug-in. The EwE discard rate represents discards for a variety of reasons including catches beyond quota. The MSE Plugin discard rate is calculated dynamically and represents only catches beyond quota. Summing the two would result in a double accounting of discards due to catches beyond quota. Because there is no way of knowing what proportion of the EwE discard rate relates to catches beyond quota, we have no way of knowing what to subtract from the summation of both rates to prevent this.

Because of this a pragmatic decision was made to use only the EwE discard rate for any years where the total catch is below quota, and use the MSE dynamic discard rate for any years where the total catch is above quota. This has the slight effect of overestimating discards when catches are below quota and underestimating discards when catches are above quota. In further work, we hope to address this issue.

#### 3.5.3 ‘Selective Fishing’ (=Avoiding discarding)

The selective fishing is a discard prohibiting policy, which mimics the fleet being able to avoid catching species beyond their allocated quota, by whatever means. Fleets target the highest value quota (as above) and can continue fishing until this species’ quota is exhausted. The ability to be selective only applies to species that have an HCR. Therefore if avoiding the catch of a species that does not typically get assigned a quota is desired, then a HCR with a zero F must be applied to that species.

Step-by-step explanation

For each fleet that has a quota for at least one functional group:

1. Determine which functional group has the highest value quota
2. Calculate the effort that would land the highest value quota

| $E_{i,j}^{T}=\frac{q_{i,j}q_{i}^{T}}{m_{i,j}^{l}M_{i}F_{i,j}B_{i}}$ | Equation 19 |
| --- | --- |

1. Calculate the effort required to catch the conservation quota for this highest value group if a conservation quota exists:

| $E_{i,j}^{C}=\frac{q_{i,j}q_{i}^{C}}{m_{i,j}^{l}M_{i}F_{i,j}B_{i}}$ | Equation 20 |
| --- | --- |

1. If conservation quota exists:

| $E_{j}=\min_{} \left( E_{i,j}^{T}, E_{i,j}^{C} \right),$  else $E_{j}=E_{i,j}^{T}$ | Equation 21 |
| --- | --- |

1. If the effort is outside the upper and lower limits reset the effort to the breached limit (see section *Determine relative effort limits for the year*)
2. For each functional group for which the fleet has a quota:
   1. Estimate the catch if the fleet was to fish at the current level for 12 months

| $C_{i,j}=E_{j}M_{i}F_{i,j}B_{i}$ | Equation 22 |
| --- | --- |

- 1. if the Catch is greater than the smallest quota from either the conservation and target HCR/prop landed as set in EwE then
     1. Set the proportion landed to quota/catch
     2. Set the proportion of the catch that is discarded and dies to:

| $m_{i,j}\left( d \right)=(1-m_{i,j}^{l})\times\frac{C_{i,j}^{L}}{C_{i,j}^{S}}\times(1-s_{i,j})$ | Equation 23 |
| --- | --- |

**Where** $m_{i,j}\left( d \right)$ is the proportion of the catch that dies, $C_{i,j}^{L}$ is the catch required to land the quota, $C_{i,j}^{S}$ is the catch that would have been caught if the fleet had not altered its mortality rate by being selective.

**Note:** The equation for the ProportionCatchDying has the effect of making the difference between the actual catch and CatchifNotSelective to all be discarded and survive, essentially as if it had never been caught. This is not an intuitive approach, but accurately represents the fleet avoiding catching a species once it has landed its entire quota.

- 1. If the catch is not greater than the smallest quota from either the conservation and target HCR/prop landed as set in EwE then
     1. Set the proportion landed to the proportion in the EwE model
     2. Set the proportion surviving being discarded to the survival proportion sampled for the given model.

Note that in all these cases the quota is the quota as calculated at the beginning of each year; it is not the remaining quota after catches and therefore does not decrease throughout the year. Consequently the relative effort calculated using this quota is the effort that will, based on the biomass and density dependent catchability at the current time-step, catch a proportion of 1/12 (since each time step represents a month) of the quota. Applying these calculated efforts at each time-step throughout the year will catch the entire quota except for when the effort is unable to adequately change due to the maximum change in the effort.

#### 3.5.4 ‘NONE’

Fleets regulated by ‘None’ have their effort maintained at a constant level equal to that at the end of the hindcast. Proportions surviving discarding are as sampled (see section 2).

#### 3.5.5. ‘No Fishing’

Fleets with this regulation setting do not fish throughout the entire projection period. This is achieved by setting the efforts for these fleets to zero.

### **Add implementation error**

Typically the historic difference between catch and quota is used to set implementation error as a proxy. Since fleet behaviour, the regulation settings, conservation quota’s and limits to changes in effort already represent many of the mechanisms that create implementation error, a value of zero was used.

Each regulation method produces implementation error for different reasons because fleets respond differently to them. In the case of the “highest value regulation” catches can be less than quota because applying effort to catch the highest value species does not fully utilise another species quota. In other cases though, it can lead to catches exceeding quota because catching the highest value quota results in catching more than the quota of another species. The “weakest stock regulation” tends to lead to underfishing in most cases, because fleets are only able to catch the entire quota of a single species. Similarly, the “selective regulation” also tends to lead to underfishing, because fleets are restricted from landing beyond quota. However, when regulated by either the “weakest stock regulation” or the “selective regulation” for some species there can be slight overfishing. This happens because a small amount of discarding is allowed to occur, meaning fleets need to catch slightly beyond quota to land up to quota (Platts & Mackinson section 5.3).

Note that there are some slight inconsistencies in the way that proportions of the catch discarded are calculated for each regulation method. These inconsistencies are rooted in the discard proportions specified in the EwE model, the discard proportions calculated by the plug-in and the problem of combining them. There are various reasons that a fleet discards: no remaining quota, undersized catch and high-grading being the most significant causes. The values in the EwE model specify as a fixed value the proportion discarded for all these reasons. The plug-in builds on this by calculating the discards dynamically, however this calculation is only of the discards due to no remaining quota. The inconsistencies arise because of the difficultly of combining these two discard proportions in an attempt to account for all elements of discarding while trying to avoid double accounting (Platts & Mackinson section 5.3).

### **Handling applied forcing F time series**

When forcing F time series are applied in the hindcast of an ecosim model it ends abruptly at the beginning of the forecast, which creates a technical problem for matching a smoothly transition in F. The problem is that the forcing F values cannot be created by multiplying the baseline catchabilities by any combination of fleet efforts. The reason for this is that the forcing Fs commonly reflect changes in the catchabilities and therefore the baseline catchabilities multiplied with the efforts no longer align with them. To solve this problem up-to-date catchabilities must be estimated, but to do this we must make an assumption about how the forced F is partitioned into fleet Fs. The assumption made is that:

| $F_{i,j}^{*}=\frac{F_{i,j}}{\sum_{j} F_{i,j}}F_{i}^{T}$ | Equation 24 |
| --- | --- |

Where $F_{i,j}^{*}$ is the partial F which is used to determine an up-to-date estimate of catchability, $F_{i,j}$ is what the F would have been on group *i* by fleet *j* if the effort at the last time step of the hindcast was applied with the baseline catchability and $F_{i}^{T}$ is the F at the forcing F at the last time step of the hindcast.

Since landings and discards can now be calculated on a per fleet basis, updated catchabilities can be calculated using:

| $q_{i,j}=\frac{L_{i,j}+D_{i,j}}{B_{i}E_{j}}$ | Equation 25 |
| --- | --- |

Where $q_{i,j}$ is the catchability,$L_{i,j}$ and $D_{i,j}$ are the landings and discards respectively of the last time step of the hindcast, $B_{i}$ is the biomass at the last time step of the hindcast and $E_{j}$ is the effort at the last time step of the hindcast.

### **Screening the Results**

Before the results of a model are saved to file, a test is performed to check whether the results are plausible. To do this the biomass trajectories are checked to be within upper and lower biomass bounds. Any model that contains a functional group with a biomass outside these bounds for any strategy has its results discarded.

**Table A. Species and their management and conservation reference points applied in the model strategies**

| NSMAP Group | **Species** | Fmp | Fmsy | Low Fmsy | High Fmsy | MSY Btrigger (t) | Lower Limit (t) Blim | Source |
| --- | --- | --- | --- | --- | --- | --- | --- | --- |
| 1 | Cod | 0.4 | 0.33 | 0.22 | 0.49 | 165,000 | 118,000 | NSMAP Annex |
| 1 | Whiting | 0.3 | 0.15 | 0.14 | 0.34 | 250,000 | 184,000 | Table 10.1 WKMSYREF32014 |
| 1 | Haddock |  | 0.37 | 0.25 | 0.52 | 88,000 | 63,000 | NSMAP Annex |
| 1 | Saithe | 0.3 | 0.32 | 0.20 | 0.43 | 200,000 | 106,000 | NSMAP Annex |
| 3 | Hake | 0.25 | 0.24 | 0.24 | 0.24 | 7,300 | 5,200 | [http://www.ices.dk/sites/pub/Publication%20Reports/Advice/2012/2012/hke-nrth.pdf . ICES hake advice plots the F and SSB trajectories cross Bpa and F pa in 2008. We use the 2008 model biomass as Bpa reference point and calculate the corresponding model equivalent to the hake advice for Blim](http://www.ices.dk/sites/pub/Publication%20Reports/Advice/2012/2012/hke-nrth.pdf) |
| 3 /7 | Norway pout |  | 0.35 | 0.35 | 0.35 | 150,000 | 90,000 | [http://www.ices.dk/sites/pub/Publication%20Reports/Advice/2013/2013/nop-34%20oct.pdf. Fmsy based on possible management plan ICES 2011.](http://www.ices.dk/sites/pub/Publication%20Reports/Advice/2013/2013/nop-34%20oct.pdf.%20Fmsy%20based%20on%20possible%20management%20plan%20ICES%202011.) |
| Pelagic | Herring | 0.26 | 0.33 | 0.33 | 0.33 | 1,000,000 | 800,000 | Table 10.3 WKMSYREF32014 |
| Pelagic | Sprat |  | 0.36 | 0.36 | 0.36 | 142,000 | 90,000 | http://www.ices.dk/sites/pub/Publication%20Reports/Advice/2013/2013/spr-nsea_201305211647.pdf |
| Pelagic | Mackerel |  | 0.22 | 0.22 | 0.22 | 85,800 | 52,624 | [http://www.ices.dk/sites/pub/Publication%20Reports/Advice/2012/2012/mac-nea.pdf. Adjusted based on fraction of NEA stock in North Sea (ICES 2016 key run report)](http://www.ices.dk/sites/pub/Publication%20Reports/Advice/2012/2012/mac-nea.pdf.%20Adjusted%20based%20on%20fraction%20of%20NEA%20stock%20in%20North%20Sea%20(ICES%202016%20key%20run%20report)) |
| Pelagic | Sandeels |  | 0.24 | 0.24 | 0.24 | 510,000 | 330,000 | [http://www.ices.dk/sites/pub/Publication%20Reports/Advice/2013/2013/san-34.pdf. Sandeel sum across areas.](http://www.ices.dk/sites/pub/Publication%20Reports/Advice/2013/2013/san-34.pdf.%20Sandeel%20sum%20across%20areas.) |
| 1 | Plaice | 0.3 | 0.19 | 0.13 | 0.27 | 230,000 | 160,000 | NSMAP Annex |
| 1 | Sole | 0.2 | 0.20 | 0.11 | 0.37 | 37,000 | 26,300 | NSMAP Annex |
| 3 | Megrim |  | 0.33 | 0.33 | 0.33 | 9,740 | 5,844 | Table 10.1 WKMSYREF32014 |
| Pelagic | Blue whiting |  | 0.32 | 0.32 | 0.32 | 22,500 | 15,000 | Assessment in 2016 ICES and ref points based on proportion of Nsea catch |
| 3 | Monkfish |  | 0.29 | 0.29 | 0.29 | 27,000 | 26,948 | Blim = 20% of the unfished biomass determined from full compensation equilibrium assessment (previous tab) |
| Pelagic | Horse mackerel |  | 0.28 | 0.28 | 0.28 | 120,000 | 119,233 | Values are given in Table 10.1 WKMSYREF3 but are very low and reflect the whole horse mackerel stock. For internal model consistency, we use the model value. |
| 3 | Dab |  | 0.28 | 0.28 | 0.28 | 373,000 | 372,208 | Blim = 20% of the unfished biomass determined from full compensation equilibrium assessment (previous tab) |
| 3 | Lemon sole |  | 0.39 | 0.39 | 0.39 | 22,000 | 21,484 | Blim = 20% of the unfished biomass determined from full compensation equilibrium assessment (previous tab) |
| 3 | Turbot |  | 0.29 | 0.29 | 0.29 | 14,000 | 13,480 | Blim = 20% of the unfished biomass determined from full compensation equilibrium assessment (previous tab) |
| 2 | Nephrops (Av. FU6-9) Expl rate |  | 0.10 | 0.08 | 0.10 | 26,030 | 20,895 | NSMAP Annex, Average over FU values. Abundance converted to biomass based on mean weight 20g (http://ices.dk/sites/pub/Publication%20Reports/Expert%20Group%20Report/acom/2015/IBP%20Neph/IBPNeph_2015.pdf) |
| 6 | Starry ray + others |  | NA | NA | NA | 45,000 | 44,918 | Blim = 20% of the unfished biomass determined from full compensation equilibrium assessment (previous tab) |
| 6 | Thornback & Spotted ray |  | NA | NA | NA | 12,000 | 11,664 | Blim = 20% of the unfished biomass determined from full compensation equilibrium assessment (previous tab) |
| 6 | Skate + cuckoo ray |  | NA | NA | NA | 13,000 | 12,161 | Blim = 20% of the unfished biomass determined from full compensation equilibrium assessment (previous tab) |

##### Table B. Specification of the management strategies

|  |  | **Dimension of the proposed NSMAP** | | | |  |
| --- | --- | --- | --- | --- | --- | --- |
| **Model strategy name** | **Category** | ***Discard scenario*** | ***Fishing mortality*** | ***Safeguard HCR*** | ***Timing*** | **Critical comparison** |
| **8 NSMAP_2015 TargetF_Weakest stock** | NSMAP | **Strict no discards:** CFP Landing obligation - fleets have to stop fishing when the quota of the 'weakest stock' is reached. | Target F = Fmsy as defined for Group 1, 2 stocks and all other assessed stocks. | Biomass conservation safeguards applied through HCRs specifying how the target F applied to each stock changes with biomass level. Four types of HCR shapes are considered. Type 1 - ICES standard, Type 2 -Precautionary, Type 3 - Protective, Type 4 - Realistic. | Target F to be achieved by 2015. | with 9 to show effect of moving to Fmsy immediately rather than slowly (by 2020) |
| **9 NSMAP 2020_TargetF_Weakest stock** | NSMAP | As in 8 | As in 8 | As in 8 | As in 8 but Target F to be achieved by 2020. | with 8 to show effect of moving to Fmsy slowly (2020) when safeguards are included. With 10&11 for effect of Low and High F ranges and 12&15 for effect of regulations |
| **10 NSMAP 2020_LowF_Weakest stock** | NSMAP | As in 8 | As in 8 but Target F = Lower F values | As in 8 | As in 9 | with 9 for effect of Low F range and 12&13for effect of regulations |
| **11 NSMAP 2020_HighF_Weakest stock** | NSMAP | As in 8 | As in 8 but Target F = Upper F values | As in 8 |  | with 9 for effect of High F range and 12 for effect of regulations |
| **12 NSMAP 2020_TargetF_Highest value** | NSMAP | **Discarding continues:** Fleets continue to discard any catches beyond the quota until all quotas are fulfilled. | As in 8 | As in 8 | As in 9 | with 9 for effect of discard regulations |
| **15 NSMAP 2020_TargetF_Selective** | NSMAP (*most realistic*) | **Avoiding discarding:** by avoiding catching fish for which there is no quota means fleets continue to fish but do not overshoot quota. | As in 8 | As in 8 | As in 9 | with 9 for effect of discard regulations |
| **23 SaveRays_Blim_no discards_Weakest stock** | NSMAP_ conservation constraint | As in 8 | Target F values defined in the NSMAP are used as the reference point for each species. But on top of that Conservation F HCRs are used (see immediate right) | The F on the target species is contingent upon the biomass of ‘conservation species’ - all the rays and skates groups. Used to represent changes to fishing when conservation species are threatened by the process of fishing activity on particular species. (e.g. dolphin bycatch). The Blim trigger point of 20% of unfished equilibrium biomass (from EwE) is used for the three skate and ray groups. Thus conservation species biomass determines the fishing rate on other species. | NA | with 9 to compare the effect of a policy where cod conservation is calling the shots |
| **6 CFP_2020 TargetF_Weakest stock** | CFP 2020 | As in 8 | As in 8 | No biomass conservation safeguards | Target F to be achieved by 2020. | with 9 to show the effect of including conservation safeguard. |
| **18 CodGod_Blim_no discards_Weakest stock** | Cod GOD | As in 8 | Target Fmsy values are used as the reference point for each species, but on top of that **conservation HCRs** are used to make the F on the target species contingent upon the biomass of ‘conservation species’. | Here we conceive that cod is the conservation species, so Fs for haddock and whiting and all others are adjusted to achieve the desired biomass of cod. The Blim for cod is used as the point at which the F rate on other species starts to decline linearly. Thus cod biomass determines the fishing rate on other species. | NA | with 9 to compare the effect of a policy where cod conservation is calling the shots |
| **20 MgtPlan_yes discard_Highest value** | Management Plans | As in 12. | Existing F targets from agreed management plans are used to define the HCRs, assuming they stay in force. Fleets discard any catches that are over quota. This represents the present situation of the previous CFP. | As in 8 | NA | with 15 for comparison with most likely change |
